# Evolution of *MIR168 *paralogs in Brassicaceae

**DOI:** 10.1186/1471-2148-9-62

**Published:** 2009-03-23

**Authors:** Silvia Gazzani, Mingai Li, Silvia Maistri, Eliana Scarponi, Michele Graziola, Enrico Barbaro, Jörg Wunder, Antonella Furini, Heinz Saedler, Claudio Varotto

**Affiliations:** 1 Environment and Natural Resources Area, Fondazione Edmund Mach, via Mach 1, 38010 San Michele all'Adige (TN), Italy; 2 Department of Science and Technology, University of Verona, strada le Grazie 15, 37134 Verona, Italy; 3 Immuno-hematology and Transfusion Service, Santa Chiara Hospital, Largo Medaglie D'Oro 9, 38100 Trento, Italy; 4 Department of Molecular Plant Genetics, Max Planck Institute for Plant Breeding Research, Carl-von-Linné-Weg 10, 50829 Cologne, Germany

## Abstract

**Background:**

In plants, expression of ARGONAUTE1 (AGO1), the catalytic subunit of the RNA-Induced Silencing Complex responsible for post-transcriptional gene silencing, is controlled through a feedback loop involving the miR168 microRNA. This complex auto-regulatory loop, composed of miR168-guided AGO1-catalyzed cleavage of *AGO1 *mRNA and AGO1-mediated stabilization of miR168, was shown to ensure the maintenance of AGO1 homeostasis that is pivotal for the correct functioning of the miRNA pathway.

**Results:**

We applied different approaches to studying the genomic organization and the structural and functional evolution of *MIR168 *homologs in Brassicaeae. A whole genome comparison of Arabidopsis and poplar, phylogenetic footprinting and phylogenetic reconstruction were used to date the duplication events originating *MIR168 *homologs in these genomes. While orthology was lacking between Arabidopsis and poplar *MIR168 *genes, we successfully isolated orthologs of both loci present in Arabidopsis (*MIR168a *and *MIR168b*) from all the Brassicaceae species analyzed, including the basal species *Aethionema grandiflora*, thus indicating that (1) independent duplication events took place in Arabidopsis and poplar lineages and (2) the origin of *MIR168 *paralogs predates both the Brassicaceae radiation and the Arabidopsis alpha polyploidization. Different phylogenetic footprints, corresponding to known functionally relevant regions (transcription starting site and double-stranded structures responsible for microRNA biogenesis and function) or for which functions could be proposed, were found to be highly conserved among *MIR168 *homologs. Comparative predictions of the identified microRNAs also indicate extreme conservation of secondary structure and thermodynamic stability.

**Conclusion:**

We used a comparative phylogenetic footprinting approach to identify the structural and functional constraints that shaped *MIR168 *evolution in Brassicaceae. Although their duplication happened at least 40 million years ago, we found evidence that both *MIR168 *paralogs have been maintained throughout the evolution of Brassicaceae, most likely functionally as indicated by the extremely high conservation of functionally relevant regions, predicted secondary structure and thermodynamic profile. Interestingly, the expression patterns observed in Arabidopsis indicate that *MIR168b *underwent partial subfunctionalization as determined by the experimental characterization of its expression pattern provided in this study. We found further evolutionary evidence that pre-miR168 lower stem (the RNA-duplex structure adjacent to the miR-miR* stem) is significantly longer than animal lower stems and probably plays a relevant role in multi-step miR168 biogenesis.

## Background

MicroRNAs (miRNAs) are a large class of recently discovered short non-coding RNAs (19–25 nt long) involved in post-transcriptional regulation of protein-coding genes. In plants they repress gene expression by catalytic mRNA degradation on the basis of sequence homology between the microRNA itself and a target sequence. Through this function they act as major players in the regulation of a series of fundamental processes in plant growth and development, in response to biotic and abiotic stress and in the regulation of components of the plant silencing machinery itself [1-4]. In plants, RNA polymerase II produces a long primary transcript (pri-miRNA) folded in a typical stem-loop structure [5,6] that is processed by a Dicer-like RNAse III ribonuclease (DCL1), first in a shorter miRNA precursor (pre-miRNA) and then in the miRNA:miRNA* duplex [7-9]. The miRNA:miRNA* duplex is transported to the cytoplasm and the mature miRNA is incorporated in the RNA-Induced Silencing Complex (RISC) where it drives the slicer ARGONAUTE1 (AGO1) to silence the target mRNA [5,10,11].

Plant miRNAs have been found in a wide variety of species and several miRNA families are evolutionarily highly conserved, ranging from mosses and ferns to dicots [1,12-16]. The members of each miRNA family normally retain a complete or almost complete conservation of miRNA and miRNA* sequences and of the structure formed by their pairing. Generally strong conservation constraints characterize the sequences and structure of the pre-miRNA hairpin structure, whereas the conservation constraints on loop and flanking sequences are less tight [1]. This is due to the fact that in plants miRNA processing depends on pre-miRNA structure rather than on sequence and in particular on the structure of the flanking sequences (lower stem) rather than on the mature miRNA itself [17]. A detailed analysis of miR163 biogenesis has revealed that the release of the mature microRNA requires at least three DCL1 cleavage steps spaced by 21 nucleotide intervals each, starting from the base of its unusually long lower stem [9]. Similar studies in animals have shown that structural features of the lower stem are essential for cleavage of pri-miRNA by Drosha (which acts in animals as DCL1 does in plants; [18]).

In contrast to the complexity that regulatory cascades of transcription factors can reach [8,19], plant microRNAs are organized according to a simple, two-level hierarchy: only three of them, miR162, miR168 and miR403 [20], control their own expression and that of the other miRNAs by targeting specific proteins involved in the post-transcriptional gene silencing pathway. In particular, miR168 regulates the function of all miRNAs by targeting *AGO1 *expression, therefore modulating its actual levels and consequently RISC activity [21,22]. *MIR168 *is present in a low copy number in different plant species [23,24] and in the Arabidopsis genome two *MIR168 *paralogs (*MIR168a *and *MIR168b*) are present. Only *MIR168a*, for which the primary transcript has been isolated [23], was shown to be involved in *AGO1 *post-transcriptional gene silencing in Arabidopsis. A miR168a-resistant version of *AGO1 *showed increased levels of *AGO1 *mRNA, the over-accumulation of miR168 and developmental defects partially overlapping with those observed in *dcl1*, *hen1 *and *hyl1 *mutants [25]. A complex feedback loop, involving on the one hand cleavage of *AGO1 *transcripts directed by miR168 and on the other hand stabilization of miR168 through AGO1 association, was shown to maintain AGO1 homeostasis which is pivotal for miRNA-mediated post-transcriptional gene silencing [26]. The overlapping expression patterns of *MIR168a *and *AGO1 *and the restored development and fertility in *ago1 *mutants expressing *miR168a-promoter:AGO1 *fusion support this model [26].

Despite the relevance of *MIR168a *in plant development, up to now no detailed comparative study has been carried out to characterize its evolution, nor has the function of its paralog *MIR168b *been determined. In this study we applied phylogenetic footprinting to the characterization of the genomic organization, and structural and functional evolution of *MIR168 *sets of orthologs in Brassicaceae. We found that, despite having originated before Brassicaceae radiation, *MIR168a *and *MIR168b *paralogs have been maintained, most likely as functional, throughout Brassicacea evolution, with *MIR168b *having undergone a partial sub-functionalization. We also provide evolutionary evidence that the lower stem in the pre-miRNA structure (the RNA-duplex structure adjacent to the miR-miR* stem) is significantly longer than lower stems in animals and propose the hypothesis that, similarly to mir163, it may play a relevant role in multi-step miR168 biogenesis.

## Results

### Synteny of *MIR168a *and *MIR168b *loci in *A. thaliana *and *P. trichocarpa*

In the genomes of both *A. thaliana *(Ath) and *P. trichocarpa *(Ptc) two *MIR168 *loci have been identified, called *MIR168a *and *MIR168b*, located respectively on chromosome 4 and 5 in Arabidopsis and on linkage_group_III and scaffold_86 in poplar [8,27].

**Figure 1:**
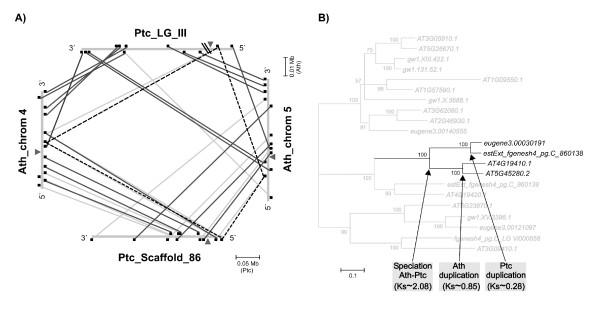
**Synteny conservation and duplication dating of *MIR168 *paralogs**. A) Synteny conservation of the genomic regions encompassing *MIR168a *and *MIR168b *in *A. thaliana *and *P. trichocarpa*. Arrows represent *MIR168a *and *MIR168b*; the squares represent coding genes with at least one homolog in both genomes; the black lines represent RBMs and the gray lines connect BLASTP hits with lower homology within the same syntenic regions. Dashed lines connect *At4g19410 *homologs; diagonal lines on Ptc_LG_III represent a 7 Mbp long region not syntenic to Arabidopsis. B) Phylogenetic reconstruction of *At4g19410 *homologs in the Arabidopsis and poplar genomes. The portion of the linearized tree representing the homologs of *At4g19410 *located in the same genomic regions as *MIR168a *and *MIR168b *is highlighted in black. Values at the branch roots correspond to majority rule consensus bootstrap values ≥ 50%. Ath: *A. thaliana*; Ptc: *P. trichocarpa*; Ks: number of synonymous nucleotide substitutions per synonymous site.

Analyses of synteny conservation were carried out by searching in poplar for the putative orthologs of the 20 Arabidopsis genes flanking *MIR168a *and *MIR168b *by screening for Reciprocal Best Matches (RBM) in BLASTP searches [28] (see Methods; Fig. 1A and Additional File 1). The queries from the former analyses were then used to identify recent segmental duplications (see Methods). Assuming orthology among the Arabidopsis and poplar genomic regions encompassing the *MIR168 *loci, the surrounding RBM pairs should be found mainly among the same pair of chromosomes. The uneven distribution of loci forming RBM pairs, however, indicated that the *MIR168 *loci may have been the result of independent duplication events.

### Dating of duplication events

Only two Arabidopsis paralogs formed RBM pairs in poplar (*At4g19410 *and *Eugene3.00030191*; *At5g45280 *and *EstExt_fgenesh4_pg.C_860138; *Fig. 1B). To determine the chronological order of these duplications, we carried out a phylogenetic reconstruction of all the genes that are homologous to the RBM pairs in the two genomes. The results show that the splitting of the two species predated two duplication events that took place independently in the Arabidopsis and poplar lineages. The two Arabidopsis paralogs, *At4g19410 *and *At5g45280*, displayed a rate of synonymous substitution (Ks) of 0.85, a higher value than that observed for paralogs resulting from the Arabidopsis alpha whole genome duplication [29]. The two poplar paralogs, *Eugene3.00030191 *and *EstExt_fgenesh4_pg.C_860138*, were confirmed to have diverged more recently (Ks = 0.28). The divergence between poplar and Arabidopsis homologs ranged between Ks = 1.91 and Ks = 2.33. Based on the estimated divergence time between Cleomaceae and Brassicaceae (Ks = 0.82, corresponding to about 41 million years ago [29]), this should correspond to a poplar-Arabidopsis divergence time of about 105 million years, in full agreement with the 100–120 million year range provided by previous reports [30].

This dating agrees with the observation that synteny conservation between Arabidopsis and poplar is higher than between Arabidopsis chromosomes. Taken together, these results indicate that no orthologous relationship can be inferred between Arabidopsis and poplar *MIR168 *homologs.

### Genomic characterization of *MIR168 *loci in Brassicaceae species

On the basis of these results we focused on analysis of the evolution and conservation between species of the two *MIR168 *homologs in a group of 16 Brassicaceae species (Table 1).

**Table 1:** Summary of *MIR168 *homolog isolation from Brassicaeae

		***MIR168a***		***MIR168b***	
**Species**	**Code**	**Upstream IR (Kbp)**	**Downstream IR (Kbp)**	**Upstream IR (Kbp)**	**Downstream IR (Kbp)**

*Aethionema grandiflora *Boiss & Hohen. ^b^	Agr	n.d.	2.0	1.3	1.3
*Alyssum montanum *L.	Amo	n.d.	4.5	n.d.	1.4
*Arabidopsis lyrata *(L.) O'Kane and Al-Shehbaz	Aly	n.d.	n.d.	n.d.	n.d.
*Arabidopsis thaliana *(L.) Heynh. ^a,b^	Ath	2.2	2.4	0.7	2.2
*Cardamine alpina *Willd.	Cal	n.d.	2.0	n.d.	3.5
*Cardamine flexuosa *With. ^b^	Cfl	n.d.	2.5	0.7	1.8
*Capsella grandiflora *(Fauché & Chaub.) Boiss. ^b^	Cgr	3.5	3.0	0.7	1.5
*Cardamine hirsuta *L. ^b^	Chi	n.d.	2.3	1.1	1.5
*Cardamine impatiens *L. ^b^	Cim	n.d.	2.3	0.8	2.0
*Calepina irregularis *(Asso) Thell.	Cir	3.5	2.3	1.0	1.3
*Diplotaxis tenuifolia *(L.) DC. ^b^	Dte	n.d.	1.5	0.6	0.8
*Erysimum cheiri *L. Crantz ^a,b^	Ech	4.0	3.0	0.5	2.0
*Malcolmia maritima *(L.) Ait. f. ^b^	Mma	4.0	3.0	0.5	1.5
*Pseudoturritis turrita *(L.) Al-Shehbaz ^a^	Ptu	3.5	2.5	0.5	1.8
*Rorippa austrica *(Crantz) Spach ^b^	Rau	n.d.	2.5	0.8	1.5
*Thellungiella halophila *(C.A. Mey.) O.E. Schulz ^a,b^	Tha	3.0	2.5	0.7	2.7

Amplification of the intergenic regions upstream and downstream of *MIR168a *and *MIR168b *in Brassicaceae species. IR: intergenic region; Kbp: kilo-basepair. n.d.: not determined.

^a^, ^b^: whole *MIR168a *or *MIR168b *loci obtained from the upstream to the downstream gene.

*MIR168a *and *MIR168b *homologs were amplified through a gene-to-gene amplification based on their up- and downstream genes in Arabidopsis. The intergenic region downstream of *MIR168a *was amplified from all the species with an amplification rate double than that of the upstream intergenic region (Table 1). In the case of *MIR168b *the intergenic regions were fully isolated (from the upstream to the downstream gene) in most of the species. The taxonomic distance of the single species from Arabidopsis did not significantly affect the isolation of intergenic regions.

The isolation of intergenic regions and the level of sequence conservation between species highlighted by their multiple alignments indicate: (1) general micro-synteny conservation in the regions surrounding *MIR168a *and *MIR168b *and (2) conservation of the orthologous relationship of all isolated *MIR168a *and *MIR168b *genes at the family level (Table 1).

### *MIR168a *and *MIR168b *phylogenetic footprinting

A clear phylogenetic footprint was identified in all species ~100–150 bp upstream of the mature miR168a (Additional File 2A) in correspondence with Arabidopsis *MIR168a *transcription start site (TSS; GenBank accession DQ108858.1). On the contrary, the use of different alignment programs failed to identify a highly conserved footprint corresponding to *MIR168b *TSS. The location of *MIR168b *TSS in Arabidopsis was therefore determined by sequencing 21 RACE products obtained from *pMIR168b1::GFP-GUS *transgenic lines. The 5' end of all clones mapped in three points of a region ~60–110 bp upstream of the mature miR168b proximal to a TATA-like motif (consensus ATTAAATACC) conserved in both paralogs (Additional File 2B; positions 28–51). The three TSS conformed in all cases to the TA class of dinucleotides identified by the YR Rule [31]. This poorly conserved footprint could be identified by manual editing of a multiple sequence alignment performed with clustalW, thus indicating a lower functional constraint on *MIR168b *as compared to *MIR168a *transcription.

Detailed analysis of pre-miR168a and pre-miR168b and flanking sequences revealed a considerable conservation of the pre-miRNA sequences at both loci (Additional File 2C and 2D). Both miR168 and miR168* were completely or almost completely conserved between orthologs and paralogs in all species (Additional File 2C and 2D). The ~20 bp flanking regions preceding the mature miR168 and following the miR168* showed a significant level of sequence conservation between orthologs and also, although to a lower extent, between paralogs (Additional File 2E, 2F and 2G).

A completely conserved 9 bp long motif (5'-TCAGATCTG-3') was isolated in both *MIR168a *and *MIR168b *just downstream of the pre-microRNA (Additional File 2E). Despite being a palindromic structure, it was not involved in any predicted secondary structure. Searches for this motif in the Athamap database [32] showed a high quality match with the binding site of the tobacco AGP1 transcription factor [33]. No significant over-representation of the 9 bp motif downstream of microRNA loci was detected as compared with coding genes (the P-value of a two-tailed G-test for patterns with a maximum of one mismatch was p = 0.066). An identical pattern was also detected in *MIR396a *downstream of, but at a higher distance as compared with *MIR168*. To check for over-representation of this motif in specific groups of microRNAs, 94 microRNA superfamilies were defined based on the classification of their targets. The application of random permutation resampling approach led to the identification of only one superfamily which showed an enrichment in this motif (p = 0.00016, α = 0.0036 at the 0.05 level applying the Bonferroni correction with k = 14 superfamily classes tested; see Methods). This superfamily encompasses both *MIR168 *paralogs and *MIR403*, a microRNA targeting *ARGONAUTE2 *(*AGO2*) that is a member of the *ARGONAUTE *family of slicers responsible for mRNA cleavage in PTGS.

A footprint specific to *MIR168b *was located about 25 bp downstream of the TSS (Additional File 2B; positions 85–118). The footprint matched the binding sites of *AGAMOUS LIKE 1 *(*AGL1*; AT3G58780) and *AGAMOUS LIKE 2 *(*AGL2*; AT5G15800), two MADS-box domain transcription factors involved in floral organ identity and meristem determinacy [34-36]. The presence of a 14 bp insertion in the basal species *Aethionema grandiflora *prompted us to separately consider two sub-motifs (consensus TGCCAGATAT and GGTAACTGTT). Their occurrence upstream of Arabidopsis microRNAs was not significantly over-represented compared to 5'UTRs of all Arabidopsis coding genes (p = 0.64, p = 0.54, respectively). No statistical support for their preferential occurrence in the 5' region of specific microRNA superfamilies was found at the 0.05 level (data not shown).

### Phylogenetic reconstruction of *MIR168a *and *MIR168b*

**Figure 2:**
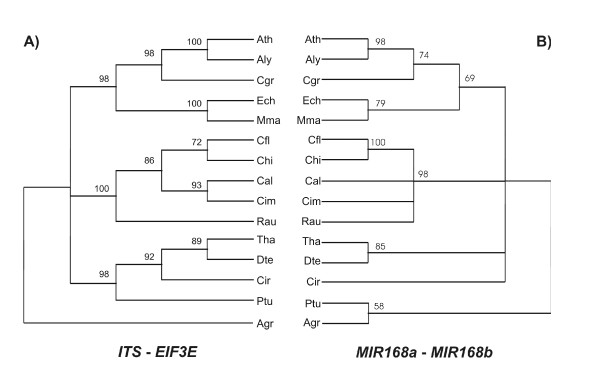
**Phylogenetic reconstruction of *MIR168 *in Brassicaeae**. Phylogenetic reconstruction of *MIR168a *and *MIR168b *in the Brassicaceae family compared with a phylogenetic tree drawn using the *IT*S and *EIF3 *markers. Values at the branch roots correspond to majority rule consensus bootstrap values ≥ 50%. A) *ITS-EIF3 *phylogenetic tree; B) *MIR168a-MIR168b *phylogenetic tree.

Phylogenetic reconstruction with all Brassicaceae *MIR168 *homologs confirmed the successful isolation of orthologs of Arabidopsis *MIR168a *and *MIR168b*. The limited amount of parsimony-informative sites, however, could not provide a phylogenetic reconstruction resolved enough to compare the evolutionary rates of the single *MIR168 *loci (data not shown). Two data partitions were created by concatenating *MIR168a *with *MIR168b *and *ITS *with *EIF3E *[37]. The resulting phylogenetic reconstructions of *MIR168 *as compared with the *ITS*-*EIF3E *neutral markers showed slightly incongruent topologies that are the consequence of the overall lower resolution provided by the *MIR168 *partition (Fig. 2).

### Comparative analysis of predicted pre-miR168a and pre-miR168b structures

**Figure 3:**
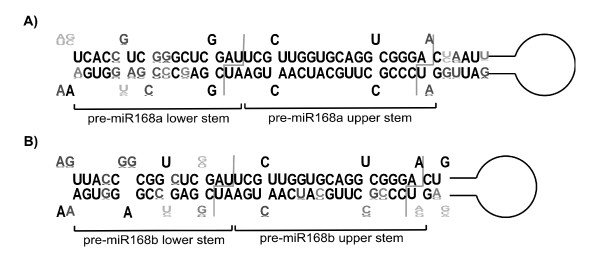
**Stem-loop structure and conservation of the *pre-miR168 *homologs**. LOGO representation of the stem-loop structure of the pre-miR168 homologs in Brassicaceae species. The base composition is indicated at each position. Gray lines correspond to the pre-microRNA processing sites. A) pre-miR168a; B) pre-miR168b.

Secondary structures for pre-miR168a and pre-miR168b plus 50 bp of flanking sequences on each side were predicted based on free energy minimization [38,39]. The consensus of the most conserved portion of these regions, including about 20 bp upstream of mature miR168 and downstream of miR168*, is shown in Figure 3. The mature microRNA-microRNA* secondary structure (upper stem) was completely conserved in the case of *MIR168a *and almost completely conserved in the case of *MIR168b *(Fig. 3A and 3B). The structure adjacent to the upper stem (lower stem) was also highly conserved in *MIR168a *and *MIR168b*. In *MIR168a *it ranged from 18 to 19 bp, with two mismatches and one bulge loop (the two mismatches typically at positions -4 and -14, the bulge loop at position -11; Fig. 3A). The lower stem of the predicted *MIR168b *structure was 17 to 18 bp long and presented three mismatches usually at positions -4, -8 and -12 (Fig. 3B). The lower stem flanking sequences distal to the upper stem were single stranded.

### Thermodynamic profiles and patterns of nucleotide substitutions

**Figure 4:**
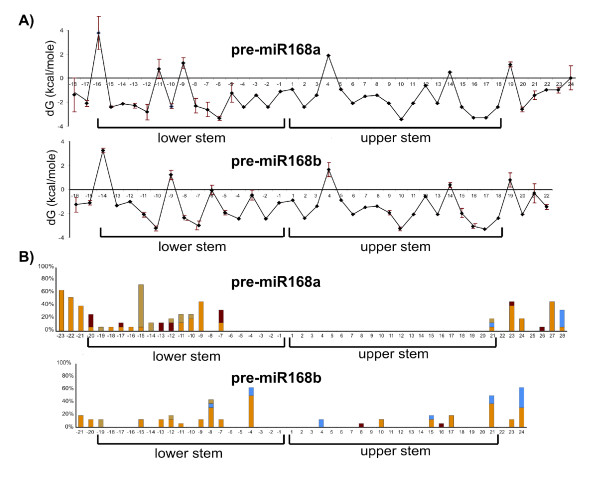
**Thermodynamic stability and nucleotide substitution profiles of pre-miR168a and pre-miR168b**. A) Thermodynamic stability profile of pre-miR168a and pre-miR168b in the Brassicaceae family. Free energy values are given in kcal/mole. Vertical bars: between-species variability calculated as double standard error. B) Distribution of nucleotide substitutions with respect to base pairing in the pre-miR168a and pre-miR168b secondary structures. Yellow: structurally conservative base substitution; ochre: base substitution comporting a change in length of a bulge loop; blue: base substitution comporting a change from unpaired to paired bases; red: base substitution comporting a change from paired to unpaired bases. The rate of nucleotide substitution is given in percentages.

The average thermodynamic profile calculated from the predicted minimum free energy (MFE) structure of each species was nearly identical at the level of the upper stem and more variable for the lower stem of both microRNAs (Fig. 4A). A common feature of both the upper and lower stem was that the secondary structure was less stable (higher free energy value, dG) at the 5' side with an increase in stability in the central part and at the 3' side. The level of nucleotidic conservation across species, however, did not correlate with the dG values, indicating that the observed footprints could not be explained by a simple increase in the stability of the corresponding secondary structure (see e.g., *MIR168a*; Fig. 4A). On the contrary, the comparison of *MIR168a *and *MIR168b *thermodynamic profiles and the classification of their nucleotide substitutions with respect to base pairing indicated a clear positional effect concerning the lower stem: the central region was more variable than the 3–4 bp close to each end of both stems. In particular the nucleotidic stretch of 5–6 bp connecting upper and lower stems of both microRNAs (position -3, +3) were extremely conserved despite having an average free energy of -1.6 Kcal/mole, which is the average free energy of both stems.

The highest number of both structurally conservative (in yellow and ochre in Figure 4B) and non-conservative nucleotide substitutions (in blue and red in Figure 4B) was found in the central portion of *MIR168a *lower stem. This was in stark contrast with the whole upper stem and the neighboring 6 bp of the lower stem in miR168a, where no nucleotide substitutions were observed, indicating the effect of a strong purifying selection. On the contrary, an overall lower number of substitutions (mostly conservative) were spread all along the stem of *MIR168b*, with a clear depletion towards the ends of both upper and lower stems.

### Expression pattern of *MIR168a *and *MIR168b*

**Figure 5:**
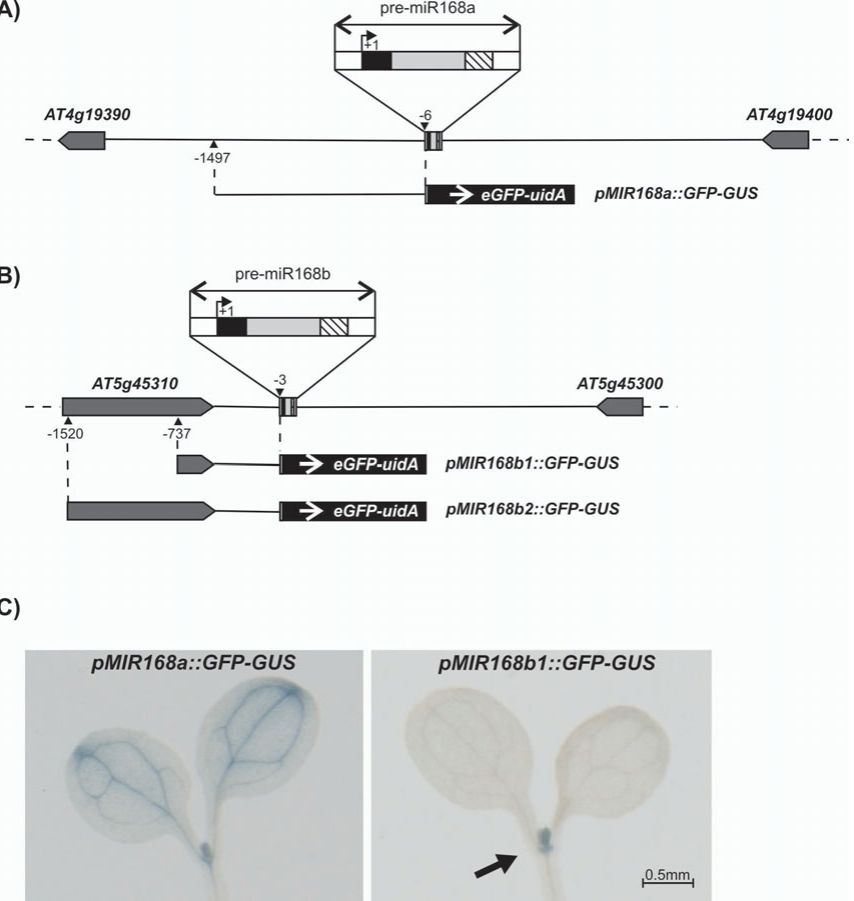
**Expression pattern of *MIR168 *paralogs in Arabidopsis**. A) Genomic region encompassing *MIR168a*; B) genomic region encompassing *MIR168b*. Black box: mature miR168; dashed box: miR168*; white boxes: 20 bp sequences forming the basal stem; light gray box: miR168 loop region; dark gray boxes: nearest exons in the genes upstream and downstream of *MIR168*, arrows indicate gene orientation. Distances are drawn to scale, with the exception of pre-miR168 (to a larger scale for clarity); +1 is the first nucleotide of the mature miR168. The *pMIR168a::GFP-GUS*, *pMIR168b1::GFP-GUS *and *pMIR168b2::GFP-GUS *constructs are represented underneath the genomic regions. C) GUS-staining of Arabidopsis transformant lines carrying the *pMIR168a::GFP-GUS *and *pMIR168b1::GFP-GUS *constructs.

The high conservation of *MIR168b *suggests that it could be expressed and functional, even though, up to now, no experimental evidence has been reported. The Arabidopsis intergenic region upstream of the mature miR168b is only approximately 500 bp long. Therefore, we used two genomic regions including the whole intergenic region plus 255 or 1038 bp upstream to functionally characterize the *MIR168b *promoter and ascertain if some regulatory elements may be present in the upstream gene. These two regions were used to drive the expression of a reporter *eGFP-uidA *fusion gene (*pMIR168b1::GFP-GUS *and *pMIR168b2::GFP-GUS*; Fig. 5B. See Methods) in stably transformed Arabidopsis transgenic lines. A construct encompassing the *MIR168a *promoter was used as a control (Fig. 5A). Both *pMIR168b1::GFP-GUS *and *pMIR168b2::GFP-GUS *constructs produced the same expression pattern (data not shown). This result indicates that the intergenic region used in the shortest construct contains all the regulatory information to drive *MIR168b *expression. Similarly to what was observed for *MIR168a*, the expression of *MIR168b *was localized in emerging leaves and in a region underneath the shoot apical meristem corresponding to leaf primordia (Fig. 5C). None of the *MIR168b *transgenic lines, in contrast to *MIR168a*, displayed expression in correspondence with vascular tissues.

## Discussion

Since the first reports about the presence of microRNAs in plants [8] a number of miRNA families have been identified. While attention has been devoted mostly to their discovery, both *in silico *and experimentally, relatively little is as yet known about plant microRNA evolution and biogenesis. In this study we applied a phylogenetic footprinting approach to the comparative study of the evolutionary patterns of two paralogous microRNA loci, *MIR168a *and *MIR168b*, in the Brassicaceae family. The presence of highly conserved phylogenetic footprints, in fact, is an indication of selective constraints acting on specific sequences [40]. If, as in the case of *MIR168*, the divergence time among genes can be demonstrated to be sufficiently high, parallel phylogenetic footprinting of paralogs provides a powerful tool to yield evolutionary evidence for the functionality of a locus as a whole or of its parts.

### Evolution of *MIR168 *in Brassicaceae

Based on the analysis of synteny conservation and on the phylogenetic reconstruction of a set of closely linked homologs, we dated the origin of Arabidopsis *MIR168 *paralogs to shortly before the divergence between the sister families Brassicaceae and Cleomaceae, about 41 million years ago [29]. Applying a genome walking method based on microsyntenic conservation, we were able to ascertain reliably the presence of and isolate both *MIR168a *and *MIR168b *paralogs in all analyzed species. The successful isolation of both *MIR168a *and *MIR168b *from the most basal crucifer, *Aethionema grandiflora*, provides demonstration that the origin of *MIR168 *paralogs predates both Arabidopsis alpha polyploidization, which took place approximately 34 million years ago (Mya) [41,42], and Brassicaceae radiation which took place between 40 and 50 Mya [43]. The limited synteny conservation observed in Arabidopsis further suggests that the *MIR168a *and *MIR168b *paralogs escaped the extensive diploidization resulting in the maintenance of only one homeolog per locus in the surrounding regions.

Similarly to *MIR319a *[44], we identified phylogenetic footprints that corresponded to functionally relevant regions, such as the TSS and the mature miR and miR* sequences, that indicate a functional conservation of both *MIR168a *and *MIR168b *throughout the Brassicacea family. Additionally, in the present study a novel 9 nt highly conserved region has been identified immediately downstream of the lower stem. The palindromic structure of this phylogenetic footprint and its pattern nearly perfectly matched the consensus-binding site of *APG1*, the tobacco putative ortholog of *A. thaliana BME3*. This would suggest its function as a homodimeric transcription factor binding site [45]. The functional complementation with *MIR168a *promoter, however, indicates that this motif is not necessary for normal *MIR168 *expression [26]. It may, instead, have a functional relevance for RNA processing or stability even while not being involved in any of the predicted pre-miR168 secondary structures. The lack of a significant over-representation downstream of other microRNA gene families in Arabidopsis indicates that this motif is not involved in a general mechanism of microRNA biogenesis or regulation. However, the occurrence of the same motif dowstream of *MIR403*, a microRNA predicted to target *AGO2 *(another member of the *AGO *family) raises the interesting possibility that it may be specifically involved in the regulation of *AGO *genes by microRNAs. Further studies are, therefore, required to clarify the functional relevance of this phylogenetic footprint.

### Role of the lower stem in miR168 biogenesis

Based on the combination of phylogenetic footprinting and secondary structure predictions, the only secondary structures conserved in *MIR168 *during the approximately 40 million years of Brassicaceae evolution were the stem containing the miR-miR* pairing (upper stem) and its distal extension (lower stem). Recently it has been demonstrated that correct animal pri-miRNA processing depends on the length of the lower stem [18]. In agreement with this, our results indicate that the lower stem is particularly conserved in *MIR168*, with the difference that the phylogenetic footprint identified in plants (ranging from 17 to 19 base pairs) is significantly longer than the 11 base pair lower stem reported for animals [18]. In animals, the Drosha-Pasha (Microprocessor) complex required for pre-miR processing is responsible for conversion of pri-miRNA to pre-miRNA [46]. In plants, this function is carried out by a functionally analogous complex involving DCL1, HYL1 and SE [47]. The observed difference in length of the lower stems may, therefore, indicate a general difference in the mechanisms of miRNA biogenesis in plants and animals.

The phylogenetic footprints identified in this study are consistent with two step pri-miRNA processing analogous to that described for *MIR163 *in Arabidopsis [9]. The recent origin of *MIR163 *and the extensive base complementarity of its inverted repeats [48] may indicate that the multi-step processing of this microRNA could be more an exception than the rule. Our finding that a clearly detectable selective pressure has been acting on *MIR168 *lower stem throughout Brassicaceae radiation indicates that multi-step pri-miRNA processing is not peculiar to *MIR163 *or to newly formed microRNAs. HYL1 has been recently shown to interact with DCL1 for the correct processing of *MIR163*. Assuming a common processing mechanism, it is possible that the highly conserved regions we identified in *MIR168 *at the ends of both lower and upper stems may be the footprints of the DCL1/HYL1 complex [49]. The phylogenetic reconstruction carried out on concatenated *MIR168a *and *MIR168b *sequences indicates that *MIR168 *evolution did not depart from that of the analyzed species. Interestingly, however, while a large difference in purifying selection is evident in *MIR168a*, the distribution of nucleotide substitutions turns out to be much more uniform in the case of *MIR168b*, as also reflected by their thermodynamic profiles. This may indicate that the lower stem has a function in fine-tuning the *pri-MIR168 *precursor processing efficiency.

### Function of *MIR168 *paralogs in Arabidopsis

The high conservation of *MIR168a *and *MIR168b *sequences, RNA predicted secondary structures and thermodynamic profiles observed in all the species we analyzed indicates that constant selective pressure has been acting on both loci throughout the Brassicaceae evolution. Interestingly, these results point to the fact that *MIR168b *has most likely been functionally conserved in all of the tested species. Former attempts to confirm *MIR168b *expression by RACE were not successful, possibly due to tissue specific expression [23,50]. In contrast to the extreme conservation observed in both *MIR168a *and *MIR319a *[44], *MIR168b *TSS identified in *A. thaliana *by RACE mapped to a phylogenetic footprint only partly conserved in the examined species, thus leaving open the possibility that the second footprint identified may function as a primary or alternative TSS in other species. This lower conservation indicates a lower selective pressure acting on the expression of *MIR168b *as compared with *MIR168a*, consistent with an accessory function of this locus [25]. However, the clear staining we observed in *A. thaliana *transformed with a *uidA *reporter gene driven by the whole intergenic regions of *MIR168b *and part of its upstream gene confirms *MIR168b *expression. Taken together, these results and the presence in the *MIR168b *stem-loop structure of the sequence information necessary for processing the mature microRNA [50], provide evidence for the functionality of this locus.

The similar but more circumscribed expression pattern of *MIR168b *as compared with *MIR168a *is consistent with either neo- or sub-functionalization of duplicated genes previously reported for other microRNA loci [51]-[52]. In light of the nearly overlapping expression patterns of *MIR168a *and *AGO1 *[26], the difference in expression in the leaf vasculature observed between *MIR168 *paralogs is most likely due to sub-functionalization of *MIR168b *than to neo-functionalization of *MIR168a*.

## Conclusion

Phylogenetic footprinting is a powerful technique for the identification of regions that, being functionally relevant, have been maintained under selective constraint during evolution [53]. We used a comparative phylogenetic footprinting approach to identify the structural and functional constraints that shaped the evolution of *MIR168 *paralogs in Brassicaceae. Previous studies in Arabidopsis demonstrated the functionality of *MIR168a *[25], but left open the possibility that *MIR168b *may be either non-functional or functionally redundant with respect to its paralog. Although their duplication happened at least 40 million years ago, we found evidence that both *MIR168 *paralogs have been maintained throughout Brassicaceae evolution. The extremely high conservation of regions functionally relevant for microRNA expression and biogenesis, predicted secondary structure and thermodynamic profile also provide evolutionary evidence of functionality of both loci, as further supported by the expression of *MIR168b *in Arabidopsis. Interestingly, the expression pattern of *MIR168b *indicates partial sub-functionalization based on the expression patterns of both *MIR168a *and *AGO1*. The identification of a highly conserved *MIR168b*-specific footprint downstream of the TSS matching the binding sites for the *AGL1 *and *AGL2 *transcription factors [34,35]-[36], provides the indication for a first candidate motif possibly involved in the regulation of *MIR168b *at specific developmental stages.

The phylogenetic footprinting carried out on the *MIR168 *paralogs finally points to the fact that the *MIR168 *lower stem (the RNA-duplex structure adjacent to the miR-miR* stem) is significantly longer than animal lower stems and possibly indicates a multistep miR168 biogenesis process analogous to the one for miR163 maturation.

The application of phylogenetic footprinting to more microRNA and plant families holds the promise of furthering our understanding of the regulation of biogenesis, the function and evolution of these intriguing regulators of both animal and plant gene expression. The design of artificial microRNAs [54,55] and its application to both basic and applied research may also greatly benefit from a more detailed identification of the determinants for efficient miRNA biogenesis.

## Methods

### Plant material

Brassicaceae species for tissue collection were grown in the greenhouse from seeds collected in Trentino Alto Adige (Italy) from wild populations or purchased from Chiltern Seeds (Bortree Stile, Ulverston, Cumbria, LA12 7PB, England. Table 1).

### Genomic isolation of *MIR168 *loci in Brassicaceae species

Genomic DNA was extracted from leaves using the CTAB method [56]. Intergenic regions encompassing *MIR168a *and *MIR168b *were obtained through gene to gene amplification by Long-Range PCR using Advantage^® ^2 Polymerase Mix (Clontech; Fig. 5A and 5B). Primers were designed either on conserved regions of the *A. thaliana *genes upstream and downstream of *MIR168a *and *MIR168b *or on the highly conserved sequences of the mature miR168 and miR168* (Additional File 3). For species where no PCR amplification was obtained, additional primers were designed on conserved sequences in the intergenic regions amplified from the other Brassicaceae species.

Amplification products were cloned in pGEM-T (Promega) or in pCR-XL-TOPO (Invitrogen) vectors. At least three clones corresponding to each product were sequenced bi-directionally to confirm their identity. *Arabidopsis lyrata *sequences were assembled from the NCBI Trace Archives . GenBank accession numbers corresponding to the sequences used in this study are provided in Additional File 4. Multiple sequence alignments were performed with M-Coffee [57] and manually edited in Bioedit [58]. Additional alignments performed with Mulan [59] were used to identify the most conserved phylogenetic footprints by using a sliding window of 5 bp and a similarity cutoff of 90%. The TSS of *MIR168b *could not be detected by means of Mulan. The results of the RACE experiments (see below) were in this case used to identify the homologous regions from the different species and the corresponding phylogenetic footprint was obtained by manual editing of multiple sequence alignments performed with ClustalW [60].

### Analysis of synteny conservation in poplar

The aminoacidic sequences corresponding to 20 Arabidopsis genes surrounding *MIR168a *and *MIR168b *(10 upstream and 10 downstream) were used for local BLASTP searches with an *e*-value cutoff of 1E-5 against the *Populus trichocarpa *genome annotation v1.1 (DoE Joint Genome Institute and Poplar Genome Consortium, . All poplar peptide homologs were used for a second BLASTP search against the Arabidopsis genome annotation v5.0 (TIGR, ). Reciprocal Best Matches (RBM, [28]) were obtained as the gene pairs with the highest E-value scores in the two analyses. To detect recent segmental duplications, an additional BLASTP search was run against a joint database containing all Arabidopsis and poplar genes using all the queries from the former analyses. The hits in the genomic regions of interest were considered if their score was better than that of any other gene from the species used as query.

### Phylogenetic reconstruction

Fast evolving nuclear loci (*ITS *[61] and *EIF3E *[37]) were used for phylogenetic reconstruction of the species used in this study. Primers used are listed in Additional File 3.

Multiple sequence alignments for the single genes obtained with M-Coffee [57] were manually refined using BioEdit [58]. PAUP* vers. 4.0 b10 [62] was used for phylogenetic analysis and tree-building using maximum likelihood (ML) with best substitution determined by Modeltest 3.7 [63]. Trees were calculated with swap = TBR, addition = random, hsearch replicates = 1000, trees hold at each step = 1, collapse = MaxBrLen, gaps were treated as missing. Bootstrapping was carried out with 100 re-sampling replicates, each performed with 100 heuristic search replicates. Phylogenetic reconstructions were carried out first on the single data partitions to assess the level of polymorphism and data congruence. Due to the low level of polymorphism in the single datasets, the partitions used for the final analyses were: 1) *ITS *+ *EIF3E*, 2) *MIR168a *+ *MIR168b*.

Phylogenetic reconstruction for the *At4g19410 *peptide homologs present in both Arabidopsis and poplar genomes was carried out with Mega 4.0 [64], using the neighbor-joining method with a variable rate among aminoacidic sites (Gamma = 1.0) and 1000 bootstrap replicates. The cladogram representing the 50% majority-rule consensus tree was used to depict the lineage divergence and duplication events. Rates of synonymous substitution (Ks) were calculated with DnaSP v4.0 [65].

### *A. thaliana *whole genome motif search

To analyze the representation of the conserved TCAGATCTG motif and of the *MIR168b*-specific footprint, the average length of the 24016 *A. thaliana *3' and 22998 5' untranslated regions (UTRs) TAIR7 blastset was calculated (233 bp and 146 bp, respectively; ). A second dataset (miRNA dataset) was obtained by extracting from the TIGR v5.0 pseudochromosomes the 233 bases downstream or the 146 bases upstream of the 184 Arabidopsis microRNA hairpins annotated in miRBase v.10.1 [66]. The presence of the TCAGATCTG motif (with a stringency of 1 mismatch) or of the two *MIR168b*-specific sub-motifs identified by the point of a 14 bp insertion in *Aethionema grandiflora *(stringency of 2 mismatches; Additional File 2B) in the miRNA and the TAIR7 3' and 5' UTR datasets was calculated with the EMBOSS fuzznuc application. A two-tailed G-test was used to test the goodness of fit for the distribution in the miRNA dataset compared with the distribution obtained from the whole genome TAIR7 UTR datasets. To check for over-representation of these motifs in specific groups of microRNAs, 94 microRNA superfamilies were defined based on classification of their targets. The number of microRNAs in each family with an occurrence of the motifs in the 233 bases downstream or the 146 bases upstream of the pre-microRNA (from now on indicated for brevity as a "hit") was further used to define 14 classes of superfamilies charcaterized by the same number of members and the same number of hits. A random permutation resampling approach was used to model the probability of each superfamily class to originate by chance in the whole complement of Arabidopsis microRNAs: a set of 1000000 random boolean strings, each 184 characters long and containing a number of "1" corresponding to the number of microRNA genes with at least one occurrence of each motif, were generated with the Mersenne Twister algorithm [67]. The probability of random occurrence of each superfamily class was given by the frequency of boolean strings matching exactly the number of hits for that class in a number of randomly selected positions corresponding to the number of its members. A Bonferroni correction was applied to keep into account multiple testing of classes.

The analysis of similarity of the conserved footprints to known binding sites was carried out by means of AthaMap database [32] and of the MultiTF program [68].

### Secondary structure prediction and thermodynamic profiles

The predicted secondary structures were generated using the RNAstructure program [38]. The LOGO representation of these structures was obtained with the WebLogo software [69].

The species-specific thermodynamic stability profiles of the predicted secondary structures were calculated for pre-miR168a or pre-miR168b according to the nearest neighbour method [39], and summarized in a single profile by averaging the free energy values at each position.

### Expression analysis of *MIR168a *and *MIR168b*

The intergenic regions upstream of miR168a and miR168b were used to drive the expression of an enhanced green fluorescent protein-beta glucuronidase (*eGFP-uidA*) fusion reporter construct (pKGWFS7; [70]). The *MIR168a *promoter region encompassed 1491 bp from -1497 to -6 upstream of the mature microRNA (Fig. 5A). For *MIR168b *two regions upstream of the mature miR, from -1520 to -3 and from -737 to -3 (including 255 and 1038 bp of the upstream gene coding sequences, respectively) were used to prepare two constructs (*pMIR168b1::GFP-GUS *and *pMIR168b2::GFP-GUS*; Fig. 5B). 4-week-old Arabidopsis plants were transformed by floral dip [71]. 15 transformed plants from each of 13 T2 lines were selected on MS medium and subjected to GUS staining [72]. Mapping of *MIR168b *TSS was carried out with the GeneRacer™ Kit (Invitrogen). Gene-specific primers are listed in Additional File 3.

## Authors' contributions

SG carried out homolog isolation, thermodynamic profile calculation and drafted the manuscript. ML isolated phylogenetic markers and participated in data analysis, made expression constructs and participated in manuscript drafting. SM and AF carried out plant transformation. ES, MG and EB participated in homolog isolation and sequencing. JW helped in phylogenetic reconstruction. HS participated in the design of the study and manuscript drafting. CV conceived and coordinated the study, took part in data analysis and drafted the manuscript. All authors read and approved the final manuscript.

## Supplementary Material

Additional file 1 **Arabidopsis-poplar *MIR168 *syntenic information. **Arabidopsis and poplar homologous gene pairs and BLASTP RBM pairs present in the *MIR168 *syntenic regions. Click here for file

Additional file 2 **Sequence alignments. **Alignment of regions containing the predicted *MIR168a *and *MIR168b *TSS, of pre-miR168a and pre-miR168b and of the region containing the conserved 9 bp motif. Click here for file

Additional file 3 **Primers. **List of primers used for amplification of *MIR168 *homologs from Brassicaceae. Click here for file

Additional file 4 **GenBank accession numbers. **List of GenBank accession numbers corresponding to the sequences obtained during this study or downloaded from public databases. Click here for file
