# Supplementary material for: Evolution of MIR168 paralogs in Brassicaceae
Source: BMC Evol Biol. 2009 Mar 23;9:62. doi: 10.1186/1471-2148-9-62 (PMC2664809; doi:10.1186/1471-2148-9-62)
Supplement: Additional file 1 — Arabidopsis-poplar MIR168 syntenic information. Arabidopsis and poplar homologous gene pairs and BLASTP RBM pairs present in the MIR168 syntenic regions. [file 1471-2148-9-62-S1.pdf]

MICROSYNTENIC PHYLOGENETIC FOOTPRINTING OF *MIR168* LOCI IN BRASSICACEAE

Silvia Gazzani, Mingai Li, Silvia Maistri, Eliana Scarponi, Michele Graziola, Enrico Barbaro, Jörg Wunder, Antonella Furini, Heinz Saedler and Claudio Varotto

Additional File 1

Arabidopsis and poplar BLASTP RBM pairs and homologous gene pairs present in the *MIR168* syntenic regions and corresponding to gray and black lines in Figure 1A, respectively.

Ath accession number	Location	Start	End	Strand	Poplar accession number	Location	Start	End	Strand	RBM
AT4G19340.1	Chr4	10559621	10560895	+	jgi|Poptr1_1|783288|fgenesh4_pg.C_scaffold_86000148	scaffold_86	1233580	1236489	-	no
AT4G19350.1	Chr4	10562034	10563768	-	jgi|Poptr1_1|297492|gw1.86.383.1	scaffold_86	1247120	1250215	+	yes
AT4G19360.1	Chr4	10564170	10565417	+	jgi|Poptr1_1|783288|fgenesh4_pg.C_scaffold_86000148	scaffold_86	1233580	1236489	-	no
AT4G19370.1	Chr4	10566161	10567849	-	jgi|Poptr1_1|827495|estExt_fgenesh4_pg.C_860147	scaffold_86	1231066	1232602	+	no
AT4G19380.1	Chr4	10568376	10572423	-	jgi|Poptr1_1|810654|fgenesh4_pm.C_scaffold_86000052	scaffold_86	1226473	1229825	+	yes
AT4G19390.1	Chr4	10574779	10576439	-	jgi|Poptr1_1|297476|gw1.86.367.1	scaffold_86	1188370	1189987	+	yes
AT4G19400.1	Chr4	10580838	10582000	-	jgi|Poptr1_1|817261|estExt_fgenesh4_pg.C_LG_III0842	LG_III	10319963	10322147	-	yes
AT4G19410.1	Chr4	10582032	10585769	-	jgi|Poptr1_1|827491|estExt_fgenesh4_pg.C_860138	scaffold_86	1136355	1139435	-	no
AT4G19410.1	Chr4	10582032	10585769	-	jgi|Poptr1_1|827492|estExt_fgenesh4_pg.C_860139	scaffold_86	1148267	1152458	-	no
AT4G19410.1	Chr4	10582032	10585769	-	jgi|Poptr1_1|553558|eugene3.00030191	LG_III	2724212	2727104	+	yes
AT4G19420.1	Chr4	10587177	10590561	-	jgi|Poptr1_1|827492|estExt_fgenesh4_pg.C_860139	scaffold_86	1148267	1152458	-	yes
AT4G19420.1	Chr4	10587177	10590561	-	jgi|Poptr1_1|827491|estExt_fgenesh4_pg.C_860138	scaffold_86	1136355	1139435	-	no
AT4G19420.1	Chr4	10587177	10590561	-	jgi|Poptr1_1|553558|eugene3.00030191	LG_III	2724212	2727104	+	no
AT4G19430.1	Chr4	10598456	10599162	-	jgi|Poptr1_1|817257|estExt_fgenesh4_pg.C_LG_III0833	LG_III	10245085	10245602	+	yes
AT4G19440.1	Chr4	10600801	10604531	-	jgi|Poptr1_1|414018|gw1.III.1121.1	LG_III	10237663	10239939	+	yes
AT4G19450.1	Chr4	10606464	10609346	+	jgi|Poptr1_1|799838|fgenesh4_pm.C_LG_III000303	LG_III	10225315	10231352	-	yes
AT4G19460.1	Chr4	10610320	10612016	-	jgi|Poptr1_1|554146|eugene3.00030779	LG_III	10223425	10224894	+	yes
AT5G45275.1	Chr5	18351632	18354607	-	jgi|Poptr1_1|799838|fgenesh4_pm.C_LG_III000303	LG_III	10225315	10231352	-	no
AT5G45280.1	Chr5	18362822	18366922	+	jgi|Poptr1_1|827492|estExt_fgenesh4_pg.C_860139	scaffold_86	1148267	1152458	-	no
AT5G45280.1	Chr5	18362822	18366922	+	jgi|Poptr1_1|827491|estExt_fgenesh4_pg.C_860138	scaffold_86	1136355	1139435	-	yes
AT5G45280.1	Chr5	18362822	18366922	+	jgi|Poptr1_1|553558|eugene3.00030191	LG_III	2724212	2727104	+	no
AT5G45290.1	Chr5	18367009	18369725	-	jgi|Poptr1_1|817260|estExt_fgenesh4_pg.C_LG_III0841	LG_III	10309691	10314860	-	yes
AT5G45300.1	Chr5	18370835	18374102	+	jgi|Poptr1_1|414016|gw1.III.1119.1	LG_III	10302181	10308051	-	yes
AT5G45310.1	Chr5	18376502	18378341	-	jgi|Poptr1_1|297474|gw1.86.365.1	scaffold_86	1175722	1178140	-	yes
AT5G45320.1	Chr5	18379338	18380395	-	jgi|Poptr1_1|810653|fgenesh4_pm.C_scaffold_86000051	scaffold_86	1184973	1185494	-	no
AT5G45320.1	Chr5	18379338	18380395	-	jgi|Poptr1_1|414318|gw1.III.1421.1	LG_III	2694005	2694556	+	yes
AT5G45330.1	Chr5	18380633	18383897	-	jgi|Poptr1_1|783288|fgenesh4_pg.C_scaffold_86000148	scaffold_86	1233580	1236489	-	yes
AT5G45340.1	Chr5	18385869	18388218	-	jgi|Poptr1_1|783291|fgenesh4_pg.C_scaffold_86000151	scaffold_86	1254410	1256451	-	no
AT5G45360.1	Chr5	18401748	18404012	-	jgi|Poptr1_1|827502|estExt_fgenesh4_pg.C_860159	scaffold_86	1326050	1329566	-	yes
AT5G45370.1	Chr5	18405518	18407647	+	jgi|Poptr1_1|836846|estExt_fgenesh4_pm.C_860060	scaffold_86	1418977	1421720	-	no
AT5G45380.1	Chr5	18408351	18413121	+	jgi|Poptr1_1|415505|gw1.III.2608.1	LG_III	10131890	10134134	+	yes
AT5G45390.1	Chr5	18413529	18415343	+	jgi|Poptr1_1|554124|eugene3.00030757	LG_III	10096309	10099359	-	yes
AT5G45400.1	Chr5	18416217	18418871	+	jgi|Poptr1_1|646356|grail3.0018008701	LG_III	10062020	10064330	-	yes
