# Supplementary material for: Evolution of MIR168 paralogs in Brassicaceae
Source: BMC Evol Biol. 2009 Mar 23;9:62. doi: 10.1186/1471-2148-9-62 (PMC2664809; doi:10.1186/1471-2148-9-62)
Supplement: Additional file 2 — Sequence alignments. Alignment of regions containing the predicted MIR168a and MIR168b TSS, of pre-miR168a and pre-miR168b and of the region containing the conserved 9 bp motif. [file 1471-2148-9-62-S2.pdf]

MICROSYNTENIC PHYLOGENETIC FOOTPRINTING OF *MIR168* LOCI IN BRASSICACEAE

Silvia Gazzani, Mingai Li, Silvia Maistri, Eliana Scarponi, Michele Graziola, Enrico Barbaro, Jörg Wunder, Antonella Furini, Heinz Saedler and Claudio Varotto

Additional File 2

A), B) Alignment of the regions encompassing *MIR168a* and *MIR168b* TSS, respectively. Arrowheads indicate transcription start sites. C) Alignment of pre-miR168a genomic region. D) alignment of pre-miR168b genomic region. E) Alignment of the sequence stretches from miR168* to the downstream 9 bp conserved motif in *MIR168a* and *MIR168b*. F), G) Alignments of *MIR168A* and *MIR168B* genomic regions from the TSS to approximatly 60 nucleotides downstream of the pre-microRNA. The alignments are reported in FASTA format and they can be visualized using programs as BioEdit Sequence Alignment Editor. In light gray are highlighted the sequences corresponding to the lower stem, while in dark grey those corresponding to the upper stem; in light blue is highlighted the 9 bp motif conserved between paralogs. For highly conserved regions dots indicate identity to the base present in the reference (first) sequence.

A)

```
                 10        20        30        40        50        60        70        80        90       100       110       120       130
        ....|....|....|....|....|....|....|....|....|....|....|....|....|....|....|....|....|....|....|....|....|....|....|....|....|....|
Ath     ATTAAATACCCTAC---AGAAGAGGCTAAGGTTGTCTCTGC-TTGATAGGGTTCCCTTCTTCTTCTTA-------TTACATATCATAAA---------------CCTCATTT---CCCCATT--ACAAAA
Agr     ATTAAATACCCAAC---AAAGAAAGCTAAA-TTTTCCCTGT-TTGATAG---ACCAATCT---------------TTACCATATTTTCA---CCATT----AACACTC-T--------------ACAAAA
Aly     ATTAAATACCCTAC---AGCTGAGGCTAAGGTTGTCTCTGC-TTGATAGGGTTCCTCCTT--------------ATTACA--TCATAAA---CCATT--CTCTGCCTCATTT---CCCCATT--ACAAAA
Cal     ATTAAATACCCTAC---ATATGAGGCTAAGGTTGTCTCTGT-TTGATAGGTTTT--------------------ATTACA--TCACAAA---CCATTTCTTTTGCCTCATTTCACCACCATTACTACAGC
Cfl     ATTAAATACCCCCTACATTATGAGGCTAAGGTTGTCTCTGT-TTGATAGGGTTC---------------------TTACA--TCACACAA--CCATTTCTTTTGCCTCATTT--CACCATTTCAACAAC-
Cgr     ATTAAATACCCTACTACAGATGAGGCTAAGGTTGTCTCTGT-TTGATAGGGTTCCTTCTTCTTCCTTCTTCCTTCTTACA--TCAATAAAAACCATT--CTTTGCCTCGTTT--CTCCCATCA-CTAAAA
Chi     ATAAAATACCCTACA--TTATGAGGCTAAGGTTGTCTCTGT-TTGATAGGGTTCTC-------------------TTACA--TCACAAAAA-CCATTC-TTTTGCCTCATTT---CACCATT--ACAACA
Cim     ATTATATACCCTACA--TTATGAGGCTAAGGTTGTCTCTGT-TTGATAGGGTTC---------------------TTACA--TCACAA----CCATTC-TTTTGCCTCATTT---CACCATT--ACAAAA
Cir     ATTAAATACCCTAC---AATAGAGGCTAAGGTTGTCTCTGT-TTCATAGGGTTCTCT------------------AAT----TCACAAA---CACT---CTTTGCCTCCTTT---CGCCATC--CCAAA-
Dte-1   AATAAATACCCTATTATACATTAGGCTAAGGTTGTCTTCGT-TTGATAGGGTTCT---------------------AAAAA-CCAGAAA---------------CACTATTT------------ACAAAA
Dte-5   AATAAATACCCTAA---ACATTAGGCTAAGGTTGTCTTCGT-TTGATAGGGTTCT---------------------AAAAA-CCAGAAA---------------CACTATTT------------ACAAAA
Ech-1   ATTAAATAGCCTAC---AGATTAGGCTTAGGTTGTCTCTGT-TCGATAGGGTTCTCTTCTC--------------TTACA--TCAGAAA---GCATT--CTTTGCCTCATTT---CGCCATT--ACAAA-
Ech-2   ATTAAATACCCTAC---AGATGAGGCTAAGGTTGTCTCTGT-TTGATAGGGTTCTCTTCT--------------CTTACA--TCAGAAA---GCATT--CTTTGCCTCATTT---CGCCATT--ACAAA-
Mma-1   ATTAAATACCCTAC---AGATGAGGCTAAGGTTGTCTCTGT-TTGATAGGGTTCTCTCTTCTCTTCTC-------TTACA--TCACAAA---CCAAT--CTTTGCCTCATTT---CGCCATT--ACAAG-
Ptu     ATTAAATACCCTAC---AAATGAAGCTAAGGTTGTCTCTGT-TTGATAGGCTTCT--------------------CTGAA--TCAAAAA---------------CTTCATTTT--CGCCATT--ACAAAA
Rau     ATTAAATACCCTAC---ATAAGAGGCTAAGGTTGTCTCTGT-TTGATAGGGTTA---------------------TTACA--TCACAA----CCATTC-TTTTACCTCACTTCTTCACCTTT--ACAAC-
Tha     ATTAAATACCCTAC----AATGAGGCTAAGGTTGTCTCAGCCCTGATAGGGTTCTT----------AAGAAAAAATATAAAATCCGAA----GCAACT-CTTTGCCTCATTT---CGTCATT--ACAAA-
```

MIR168A TSS footprint

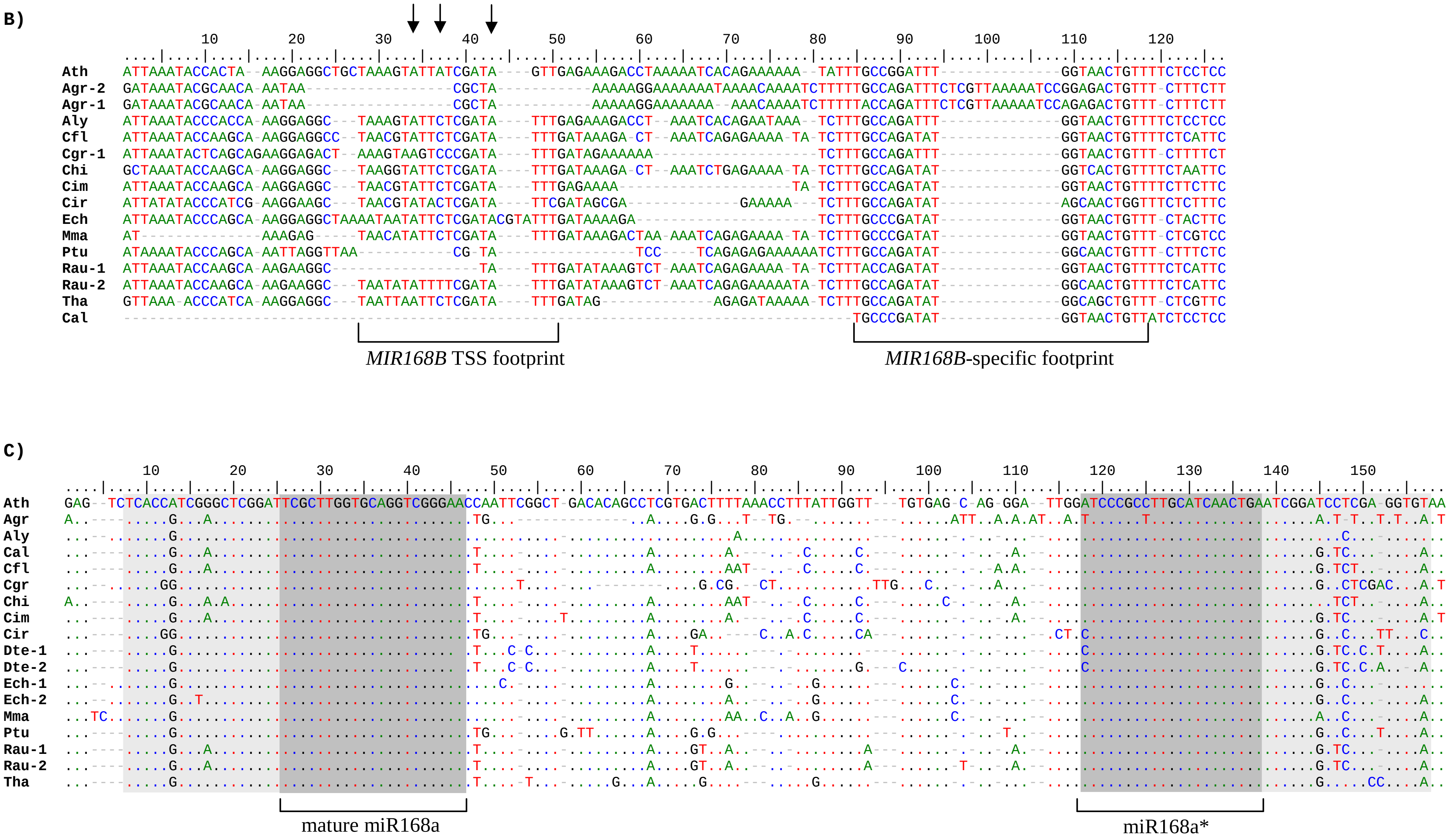
B)
Ath
Agr-2
Agr-1
Aly
Cfl
Cgr-1
Chi
Cim
Cir
Ech
Mma
Ptu
Rau-1
Rau-2
Tha
Cal
MIR168B TSS footprint
MIR168B-specific footprint
C)
Ath
Agr
Aly
Cal
Cfl
Cgr
Chi
Cim
Cir
Dte-1
Dte-2
Ech-1
Ech-2
Mma
Ptu
Rau-1
Rau-2
Tha
mature miR168a
miR168a*

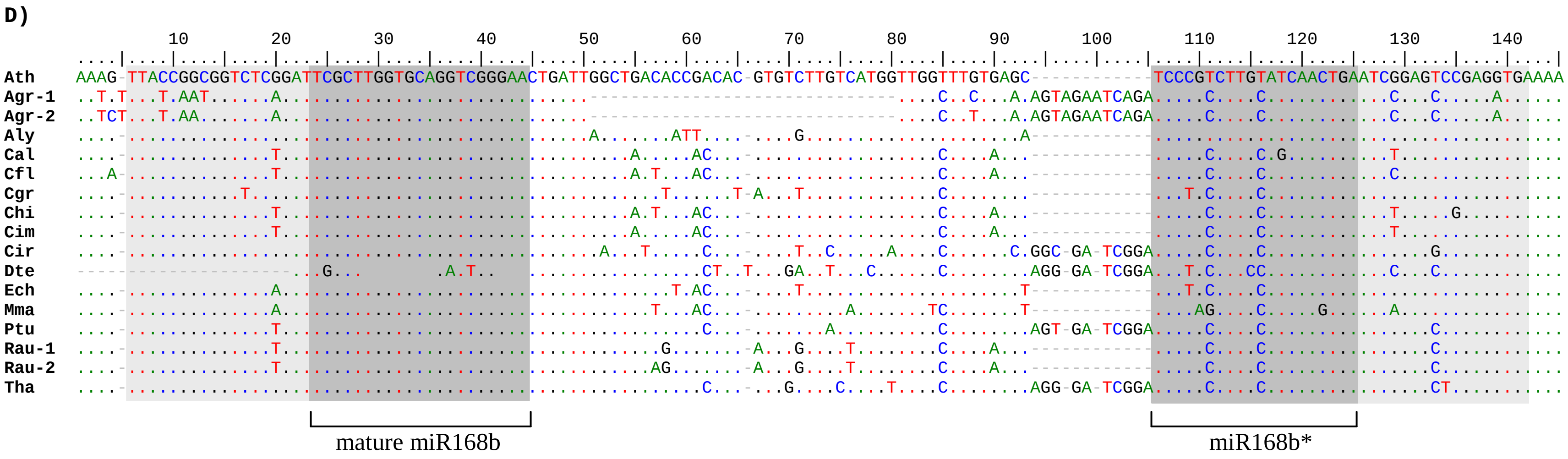
D)
Ath
Agr-1
Agr-2
Aly
Cal
Cfl
Cgr
Chi
Cim
Cir
Dte
Ech
Mma
Ptu
Rau-1
Rau-2
Tha
mature miR168b
miR168b*

E)

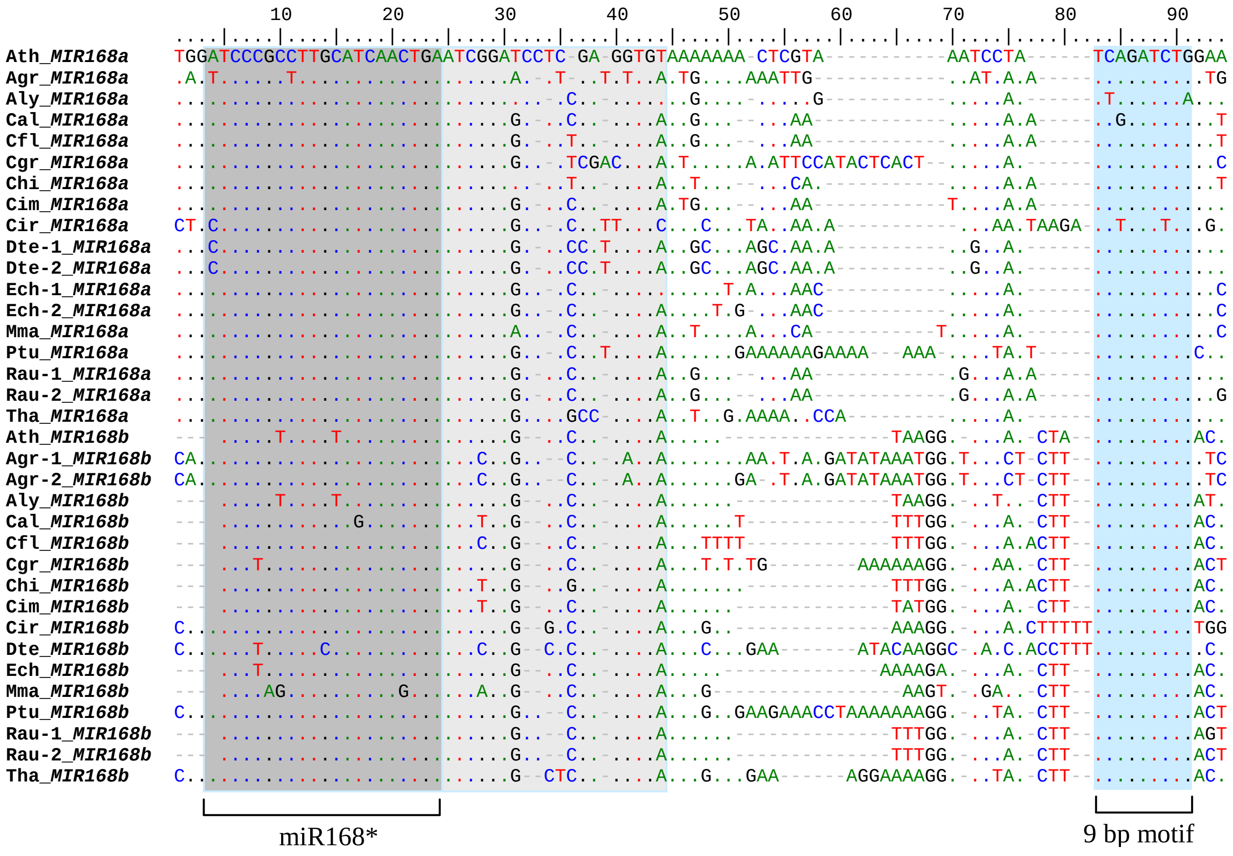

Ath_MIR168a
Agr_MIR168a
Aly_MIR168a
Cal_MIR168a
Cfl_MIR168a
Cgr_MIR168a
Chi_MIR168a
Cim_MIR168a
Cir_MIR168a
Dte-1_MIR168a
Dte-2_MIR168a
Ech-1_MIR168a
Ech-2_MIR168a
Mma_MIR168a
Ptu_MIR168a
Rau-1_MIR168a
Rau-2_MIR168a
Tha_MIR168a
Ath_MIR168b
Agr-1_MIR168b
Agr-2_MIR168b
Aly_MIR168b
Cal_MIR168b
Cfl_MIR168b
Cgr_MIR168b
Chi_MIR168b
Cim_MIR168b
Cir_MIR168b
Dte_MIR168b
Ech_MIR168b
Mma_MIR168b
Ptu_MIR168b
Rau-1_MIR168b
Rau-2_MIR168b
Tha_MIR168b
miR168*
9 bp motif

F)

>Ath_*MIR168a*
GATAGGGTTCCCT-------TCTTCTTCTTATTA-CAT-----------ATC--ATAAACCTCATTT---CCCCATTAC-AAAACCTAGA------ACTCCTTCTCTCTTCTCTTTCTTCATA------TCCCTG-------------------TCT-----------
--------AAAGGGATT------ATT-ATGATAGT--AGAG--TCTCACCATCGGGCTCGGATTCGCTTGGTGCAGGTCGGGAACCAATTCGGC-TGA-CACAGCCTCGTGACTTTTAAACCTTTATTGGTT---TGTGAGC--
AG-GGA--TTGGATCCCGCCTTGCATCAACTGAATCGGATCCTCGA-GGTGTAA----AAAA----------ACTCGTAAATCCTA-TCAGATCTG-----GAAGATT----------------TCT--ACGC--T--------T-CTCC-TTCTTT-
ATAT
>Agr-1_*MIR168a*
GATAGACCAATCT------------------TTACCATATTTT-CACCATT-----AACACTC-T------------AC-AAAACCTAAA----ACTCTTC-TCTCTCTCCTC-TTTTTTCACTTTCTCTC-------------------------TACA-------------
TAGAGAGA--------------TTGAT-----AAAG----TCACCGTCGAGCTCGGATTCGCTTGGTGCAGGTCGGGAACTGATT--------------CCACGTGGCGTTTT----TGTATTGGTT---
TGTGAGATTAGAGAAATTTAGTTCCCGCTTTGCATCAACTGAATCGGAAC-TTGATGTTGAATGAAAAAAA-------------TTGAAATCAAA-TCAGATCTGGT---GGAGATA----------------TCT-----------------
TACTC-TCTCTTT-CTTT
>Aly_*MIR168a*
GATAGGGTTCCTC--------------CTTATTACATC---ATAAACCATT--CTC-TGCCTCATTT---CCCCATTAC-AAAACCTAAA------------TCTCCTTCTCTCTTCTCTCTT------CT------------------------TCT-----------
AAAGAGAGAGAGAGAG-------ATT-ATGATAT--AAGAG--TCTCACCGTCGGGCTCGGATTCGCTTGGTGCAGGTCGGGAACCAATTCGGC-TGA-CACAGCCTCGTGACTTTAAAACCTTTATTGGTT---
TGTGAGC--AG-GGA--TTGGATCCCGCCTTGCATCAACTGAATCGGATCCCCGA-GGTGTAA----GAAA----------ACTCGTGAATCCAA-TTAGATCTA-----GAAGAAA----------------TCT--ACTC--T--------T-
CTCC-TTCTTT-ATCT
>Cal_*MIR168a*
GATAGG--------------------TTTTATTA-CATC---ACAAACCATTTCTTTTGCCTCATTTCACCACCATTAC-TACAGCTTCA--------------TCTCTTATCTTTCTTCCTCACAAAGCCTTCG--------------------
TTTCAGTTTTCTTGAGAGCGAGGGAGAGATTACG---ATAGATGAT-----AGAG----TCACCGTCGAGCTCGGATTCGCTTGGTGCAGGTCGGGAACTAATT-GGC-TGA-CACAGCCACGTGACTTAT----
CCTCATTGGCT---TGTGAGC--AG-GAA--TTGGATCCCGCCTTGCATCAACTGAATCGGAGCTCCGA-GGTGAA-----AGAA----------ACTCAAAATCCAAA-TCGGATCTG------GATGATT-------
TAGAAGAAATCT--TCTC--T--------T-TTCT-TTCTTT-TTA
>Cfl_*MIR168a*
GATAGGGTTCTT---------------------A-CATC--ACACAACCATTTCTTTTGCCTCATTT---CACCATTTC-AACAACTTCT--------CTCTTCTCTCTTATCTTTTCTTCCTCACAAGTCTTCG--------------------
TTTCAGTTTTCTTGAGAGCA-AGAAAGA-TTACG---TTAGATGAT-----AGAG----TCACCGTCGAGCTCGGATTCGCTTGGTGCAGGTCGGGAACTAATT-GGC-TGA-CACAGCCACGTGACTTAAT---
CCTCATTGGCT---TGTGAGC--A-AGAA--TTGGATCCCGCCTTGCATCAACTGAATCGGAGCTCTGA-GGTGAA-----AGAA----------ACTCAAAATCCAAA-TCAGATCTG-----GATGATT----------------TTT---------
----
>Cgr_*MIR168a*
GATAGGGTTCCTTCTTCTTCCTTCTTCCTTCTTA-CATCAATAAAAACCATT--CTTTGCCTCGTTT--CTCCCATCACTAAAACCTAAA-----GGGAGACTCCTTCTCCTCCTTATCTCTC------TCTCTG---------------
-----TCT------------------AGAGAGA-TTCTT---ATATATAAACAGCGAGAG--TCTCACGGTCGGGCTCGGATTCGCTTGGTGCAGGTCGGGAACCAATTTGGC-TGA-------CGTGAGTCGTCT----
CTTTATTGGTTTTGTGTCAGC--AGAGGA--TTGGATCCCGCCTTGCATCAACTGAATCGGAGCCCTCGACGTGAAT----AAAAAACATTCCATACTCACTAATCCAA-TCAGATCTG-----GACC-------------------
TAT--ACTCGTT--------T-CTCC-TTCTTT-TTA-
>Chi_*MIR168a*
GATAGGGTTCTCT-------------------TA-CATCA-CAAAAACCATT-CTTTTGCCTCATTT---CACCATTAC-AACACCT------------------TCCTTCTCTCTTTATCTT------TCTTCG--------------------
TTTCAGTTTCGTTGAGAGCAATAAAAAGATTTAC--GATAGATGAT-----AAAG----TCACCGTCGAGATCGGATTCGCTTGGTGCAGGTCGGGAACTAATT-GGC-TGA-CACAGCCACGTGACTTAAT---
CCTCATTGGCT---TGTGACC--AG-GAA--TTGGATCCCGCCTTGCATCAACTGAATCGGATCTCTGA-GGTGAAA----TAAA----------CTCCAAAATCCAAA-TCAGATCTG-----GATGGTT------
TTTGAAGAAATCTGAAATC--T--------T-CTCC-TTCTTT-TTAT
>Cim_*MIR168a*
GATAGGGTTCTT---------------------A-CATCA----CAACCATT-CTTTTGCCTCATTT---CACCATTAC-AAAACCT------------TCTCTCTTCTTATCTCTCTTCCTC-ACAAGTCTTCG--------------------
TTTCAGTTTTCTTGAGAGCA-AGAAAGA-TTACC---ATAGATGAT-----AGAG----TCACCGTCGAGCTCGGATTCGCTTGGTGCAGGTCGGGAACTAATT-GGCTTGA-CACAGCCACGTGACTTAT----
CCTCATTGGCT---TGTGAGC--AG-GAA--TTGGATCCCGCCTTGCATCAACTGAATCGGAGCTCCGA-GGTGAA-----TGAA----------ACTCAATATCCAAA-TCAGATCTG-----GAAGATT-------
TAGAAGAAATCT--ACTC--T--------T-CTCC-TTCTTT-CTAT
>Cir_*MIR168a*
CATAGGGTTCTCT--------------------A-ATTC---ACAAA-CACT--CTTTGCCTCCTTT---CGCCATCCC-AAA-CCTAAA-------
CTTCTTCTCTCTTCTCTTTCTTCTCTCTGGTTCTGTCTTCGTGTTCTTATTTTGTTTTCCTCAGATTTCT-------AGAGAGAGA-TTG--------TGTGAT-----AGAG----
TCACGGTCGGGCTCGGATTCGCTTGGTGCAGGTCGGGAACTGATT-GGC-TGA-CACAGCCACGTGGATT----CCCATCATTGGCA---TGTGAGC--AG-GGA--
TCTGCTCCCGCCTTGCATCAACTGAATCGGAGCCCCGATTGTGCAAA---CAAA----------TATCAAAAATCAAA-TAAGATCTGATTTGGGAGATA----------------TAT--ACTC--T------CCT-CTCC-TATTTC-ATTT
>Dte-2_*MIR168a*
GATAGGGTTCT--------------------------------AAAAACCAGAAACACTAT---------------TTAC-AAAACCTAAA-------ACTTATCTTTCTTCTCTCTCTCTTTA------TTTTTT----------CTTTTATGGCTTTCAGATTCC------
----AGAGAC-------------TATGGT-----AGAG----TCACCGTCGGGCTCGGATTCGCTTGGTGCAGGTCGGGA-CTAATC-CGC-TGA-CACAGCCACGTGTCTTTT----ACTTATTGGTT----GTGAGC--AG-GGA--
TTGGCTCCCGCCTTGCATCAACTGAATCGGAGCTCCCATGGTGAAAGC--AAAA----------GCCAAAAAAAGCCAA-TCAGATCTG-----GAAAAATATATCCTTTGTGTGTGTCT--ATGT--TGATGCATAT-CTTC-
TTCTGT-ATAT

>Ech-1_*MIR168a*
GATAGGGTTCTCT--------------TCTCTTA-CATC---AGAAAGCATT--CTTTGCCTCATTT---CGCCATTAC-AAA-CCTAAT---------TCTCCTCCCTCTCTTCTCTTTCTT------CTTCTT----------------------TAG-----------
---------------------------------AGAG--TCTCACCGTCGGGCTCGGATTCGCTTGGTGCAGGTCGGGAACCAACT-GGC-TGA-CACAGCCACGTGACTTGTA---CCTTGTTGGTT---TGTGAGCC-AG-GGA--
TTGGATCCCGCCTTGCATCAACTGAATCGGAGCCCCGA-GGTGTAAA---AATA----------ACTCAACAATCCAA-TCAGATCTG-----GACGAAA----------------TCT--ACTC--T--------TCCTCC-TTGTTT-ATAT
>Mma_*MIR168a*
GATAGGGTTCTCT-------CTTCTCTTCTCTTA-CATC---ACAAACCAAT--CTTTGCCTCATTT---CGCCATTAC-AAG-CCTC-----------TCTTCTCTTTCTTCT-TCTTCTTA------TCTCTGTGT-------------------CTAG----
---AGAGTCTCTCACCGTCGGGCTCGGATTCGCTTGGTGCAGGTCGGGAACCAATT-GGC-TGA-CACAGCCACGTGACTTAAAACCCATTGTTGGTT---TGTGAGCC-AG-GGA--
TTGGATCCCGCCTTGCATCAACTGAATCGGAACCCCGA-GGTGAAAT---AAAA----------ACTCCATAATCCAA-TCAGATCTG-----GACCATA----------------TCT--ACTC--T--------T-CTCA-TTCTTT-GTAT
>Ptu_*MIR168a*
GATAGGCTTCTCTGA---------------------ATC---AAAAA------------CTTCATTTT--CGCCATTAC-AAAACCTAAA----------CTTCTCTTTCTTCTCTCTCTGTT------T-------------------------
TTTCAGATTTCTAGTGAGAGAAGAGAGAGAGAGAG--AT-TATGAT-----AGAG----TCACCGTCGGGCTCGGATTCGCTTGGTGCAGGTCGGGAACTGATT-GGC-TGGTTACAGCCACGTGGCGTTT----
CTTTATTGGTT---TGTGAGC--AG-TGA--TTGGATCCCGCCTTGCATCAACTGAATCGGAGCCCCGATGGTGAAA----AAAAGAAA--AAAGAAAAAAAAAATCTAATTCAGATCTG-----CAAGAAA----------------
TCT--ACTC--T--------TTCTCC-TTCTTCAATAT
>Rau-1_*MIR168a*
GATAGGG---------------------TTATTA-CATC---ACAACCA--TTCTTTTACCTCACTTCTTCACCTTTAC--AACTTTCAGC------TTCTCTCTTTATCTTTTTTTCCTCAA------TCTTCG--------------------
TTCCAGTTTTCTT---------GAGAGA-TTATGA---TAGATGATA----AGAG----TCACCGTCGAGCTCGGATTCGCTTGGTGCAGGTCGGGAACTAATT-GGC-TGACACAGCCACGTGGTTTATAC---CTT-
ATTGGTA---TGTGAGC--AG-GAA--TTGGATCCCGCCTTGCATCAACTGAATCGGAGCTCCGA-GGTGAAA--------GAAA--------CTCAAAGTCCAAATCAGATCTG--------GAAGA-----TTTGAAGAAATCTT-
ACTCT-----------TCTCCTTCTTTATATTC
>Tsa_*MIR168a*
GATAGGGTTCTTAAGAAAAAATATAAA---------ATC---CGAAGCAACT--CTTTGCCTCATTT---CGTCATTAC-AAATCTAAAACTTTTGCTTCTTCTATCTTTCCTCTCTCTCTC------TCTCTG---------
TCTCTGTTTT----CGCCA--------------------------------------TT---AGAG----TCACCGTCGGGCTCGGATTCGCTTGGTGCAGGTCGGGAACTAATT-TGC-TGA-CACGGCCACGTGAGTTTTC---CTTTGTTGGTT---
TGTGAGC--AG-GGA--TTGGATCCCGCCTTGCATCAACTGAATCGGAGCCTCGCCGGTGAAA-----TAAGAAA------AAGTCCAAATCCAA-TCAGATCTG-----GAAGACA----------------TGT--ATTC--T--------T-
CTCC-TTCTTC-ATGT

G)

>Ath_*MIR168b*
ATTAAATACC-ACTA-AAGGAGGCTGCTAAAGTATTATCGATA----GTTGAGAAAGACCTAAAAATCACAGAAAAAA--TATTTGCCGGATTT--------------GGTAACTGTTTTCTCCTCCTTAT------
TCTCTTTCACCTT-TTTCTGATGAGAGAGAGA-------------------------------TGCGATGAT--TGTTA-------AAG-TTACCGGCGGTCTCGGATTCGCTTGGTGCAGGTCGGGAACTGATTGGCTGACACCGACAC-
GTGTCTTGTCATGGTTGGTTTGTGAGC------------TCCCGTCTTGTATCAACTGAATCGGAGTCCGAGGTGAAA-AAATAA---------------GGATCC-AAC---TATCAGATC--TGACACAATTATTCTGTTT-------------
------------GTTGTTCGTCTAA-------------TTTTTTTGTTGAAA
>Agr-1_*MIR168b*
GATAAATACGCAACA-AATAA-----------------CGCTA-----------AAAAAGGAAAAAAA--AAACAAAATCTTTTTACCAGATTTCTCGTTAAAAATCCAGAGACTGTTT-CTTTCTT-
TCTTTCGTTTTTGTTCCAAATCTTTTCT-TTGATTT-GGTGGA--ATGTTTATCAATGGTCTCAGATTCGCTTGGTGCAGGTCGGGAACTGATT------------------------------
TTGGCTTCTGAACAGTAGAATCAGATCCCGCCTTGCATCAACTGAATCCGAGCCCGAGATGAAAAAAAAAAATTGAAGATATAAATGGATTCC-CTC---TTTCAGATC--
TGGTCAAATCTTATATTTTTTTTCGATAATTCCAGGAAAGAATGATTGAATCTTGAATCTTGATTCATTAATCTTGCTT---AT
>Aly_*MIR168b*
ATTAAATACCCACCA-AAGGAGGC---TAAAGTATTCTCGATA----TTTGAGAAAGACCT--AAATCACAGAATAAA--TCTTTGCCAGATTT--------------GGTAACTGTTTTCTCCTCCTTAT-----
TATCTTTCACCTTTTTTCCAGT-GAGCGATAGAGAGAGAAA-----------------------TACGATGAT----TTA-------AAG-
TTACCGGCGGTCTCGGATTCGCTTGGTGCAGGTCGGGAACTGATTAGCTGACAATTACAC-GTGTGTTGTCATGGTTGGTTTGTGAGA------------
TCCCGTCTTGTATCAACTGAATCGGAGTCCGAGGTGAAAAAAATAA---------------GGATCT-TAC---TTTCAGATC--TGATACAATCTTTTTTT------------------------GCGGTAGAATCTTGAA-------------
AACTTTTTGTTTAT
>Cal_*MIR168b*
---TGCCCGATAT--------------GGTAACTGTTATCTCCTCCTTCTTCTTCTTCTCTTTCATCTT--
TTCCAATGAGAGAAATAGAGAAAGAAAAAAAAAG--------------TACGATGAT---GATA-------AAG-TTACCGGCGGTCTCTGATTCGCTTGGTGCAGGTCGGGAACTGATTGGCTAACACCACCAC-
GTGTCTTGTCATGGTTGGCTTGTAAGC------------TCCCGCCTTGCAGCAACTGAATCTGAGTCCGAGGTGAAAAAAATTTT--------------GGATCC-AAC---TTTCAGATC--TGACAC-TTTTCGTC-ACTCTT------
------------AAGGTAAAATCTTGAT-------------ATTTTGTTT---GT
>Cfl_*MIR168b*
ATTAAATACCAAGCA-AAGGAGGCC--TAACGTATTCTCGATA----TTTGATAAAGA-CT--AAATCAGAGAAAA-TA-TCTTTGCCAGATAT--------------GGTAACTGTTTTCTCATTCTTCT------
TCTCTTTCATCTT-TTTCCATTGAGAGAGATCGGAAAAAAAAAAAAAAAAAAAAAAAAAAAAAAGTACGATGATGATGATA-------AAA-

TTACCGGCGGTCTCTGATTCGCTTGGTGCAGGTCGGGAACTGATTGGCTAATACCACCAC-GTGTCTTGTCATGGTTGGCTTGTAAGC------------
TCCCGCCTTGCATCAACTGAATCCGAGTCCGAGGTGAAAATTTTTTT--------------GGATCCAAAC---TTTCAGATC--TGACACTTTCTTCGTT-ACTCTT------------------AAGGTTAAATTTTGAT-------------
ATTTTGTTT---AT
>Cgr-1_*MIR168b*
ATTAAATACTCAGCAGAAGGAGACT--AAAGTAAGTCCCGATA----TTTGATAGAAAAAA-------------------TCTTTGCCAGATTT--------------GGTAACTGTTTCTTTTCTCTTCTCTTTCATCTTTTTTTTTTC-
TTTCTAATGAGAAAGAGAGAAAGAGAGA---------------------TACGATGAA--GGTTACAAAAAAAAG-TTACCGGCGGTTTCGGATTCGCTTGGTGCAGGTCGGGAACTGATTGGCTGACTCCGACAT-
ATGTTTTGTCATGGTTGGCTTGTGAGC------------TCCTGCCTTGCATCAACTGAATCGGAGTCCGAGGTGAAAATATATGAAAAAA---------GGATCA-AAC---TTTCAGATC--TGACTCGATCTT-----CATCTT--
----------------ACGGTAGAATCTTTAT-------------ATTTTTTGTTGAAA
>Chi_*MIR168b*
GCTAAATACCAAGCA-AAGGAGGC---TAAGGTATTCTCGATA----TTTGATAAAGA-CT--AAATCTGAGAAAA-TA-TCTTTGCCAGATAT--------------GGTCACTGTTTTCTAATTCTTCT------
CCTCTTTCATATT-TTTCCATTGAGAGAGAATAGAGCAAAAG---------------------TACGATGAT---GATA-------AAG-
TTACCGGCGGTCTCTGATTCGCTTGGTGCAGGTCGGGAACTGATTGGCTAATACCACCAC-GTGTCTTGTCATGGTTGGCTTGTAAGC------------
TCCCGCCTTGCATCAACTGAATCTGAGTCGGAGGTGAAAAAAAAATTT--------------GGATCCAAAC---TTTCAGATC--TGACACGTTCTTCGTTCACTCTT------------------AAGGTTAAATATTGAT-------------
ATTTGGTTT---AT
>Cim_*MIR168b*
ATTAAATACCAAGCA-AAGGAGGC---TAACGTATTCTCGATA----TTTGAGA-------------------AAA-TA-TCTTTGCCAGATAT--------------GGTAACTGTTTTCTTCTTCTTCTTCT---TCTCTTTCATCTT--
TTCCATTGAGAGAGATAGAAAAAAAAAAAAAAAG---------------TACGATGAT---GATA-------AAG-TTACCGGCGGTCTCTGATTCGCTTGGTGCAGGTCGGGAACTGATTGGCTAACACCACCAC-
GTGTCTTGTCATGGTTGGCTTGTAAGC------------TCCCGCCTTGCATCAACTGAATCTGAGTCCGAGGTGAAAAAAAATAT---------------GGATCC-AAC---TTTCAGATC--TGACACATTCTTCGTC-ACTCTT-----
-------------AAGGTTAAATCTTGAT-------------ATTTTGTTT---AT
>Cir_*MIR168b*
ATTATATACCCATCG-AAGGAAGC---TAACGTATACTCGATA----TTCGATAGCGA-------------GAAAAA---TCTTTGCCAGATAT--------------AGCAACTGGTTTCTCTTTCTTCTCT----
TTTGTTTTATCTTTTTCCAATGACGGAAAGAGAGAGA-GA-----------------------GACGATGATGATGGTA-------AAG-
TTACCGGCGGTCTCGGATTCGCTTGGTGCAGGTCGGGAACTGATTGACTGTCACCGCCAC-GTGTTTTCTCATGATTGGCTTGTGACCGGCGATCGGA--
TCCCGCCTTGCATCAACTGAATCGGAGGCCGAGGTGAAAAGAAAAA---------------GGATCC-AACTTTTTTCAGATCTGTGGCTCAATCTTTCTTGACTCTT------------------AAGGTAGAAGCTTGAA-------------
ATCTTGTTT--AAC
>Dte_*MIR168b*

CTGATTGGCTGACACCGCTACTGTGGATTTTCACGGTTGGCTTGTGAGCAGGGATCGGA--TCCTGCCTTCCATCAACTGAATCCGAGCCCGAGGTGAAAACAAAGAAATACAAG-------GCTACC-AAC-
CTTTTCAGATC--TGGC-------------A-----------------------------------TGAA-------------ACCTTTTTT---TT
>Ech_*MIR168b*
ATTAAATACCCAGCA-AAGGAGGCTAAAATAATATTCTCGATACGTATTTGATAAAAGA---------------------TCTTTGCCCGATAT--------------GGTAACTGTTT-CTACTTCTTT--------CTCTTACATCTT-
TTTCCAATGAGAGACAGAGAGAGAGA-----------------------TACGATGAT--GGTTA-------AAG-TTACCGGCGGTCTCAGATTCGCTTGGTGCAGGTCGGGAACTGATTGGCTGACATCACCAC-
GTGTTTTGTCATGGTTGGTTTGTGAGT------------TCCTGCCTTGCATCAACTGAATCGGAGTCCGAGGTGAAAAAAAAAAA---------------GAATCC-AAC---TTTCAGATC--TGACAT-----------CCTCTT---------------
---ATGGTACAATCTTGAA-------------ATTTTTTTT---AT
>Mma_*MIR168b*
AT--------------AAAGAG-----TAACATATTCTCGATA----TTTGATAAAGACTAA-AAATCAGAGAAAA-TA-TCTTTGCCCGATAT--------------GGTAACTGTTT-CTCGTCCTTC---------TCTTTCATCTG---------
GAGATACGATGA-----------------------------TG-GATGAT--TGTTA-------AAG-TTACCGGCGGTCTCAGATTCGCTTGGTGCAGGTCGGGAACTGATTGGCTGATACCACCAC-
GTGTCTTGTAATGGTTGTCTTGTGAGT------------TCCCAGCTTGCATCAAGTGAATCAGAGTCCGAGGTGAAAAGAA------------------GTATGA-TAC---TTTCAGATC--TGACAC-----------AATCTT-----------------
-ATGGTACAATCTTGAA-------------ATTTTATTT---AC
>Ptu_*MIR168b*
ATAAAATACCCAGCA-AATTAGGTTAA-----------CG-TA----------------TCC----TCAGAGAGAAAAAAATCTTTGCCAGATAT--------------GGCAACTGTTT-CTTTCTCTTCTCTTTTTAATCTTTTTTTCTCTC-
GATGAGAGAGAAGAAAGATGAGAGA---------------------TACGATGAT---GATAA------AAG-TTACCGGCGGTCTCTGATTCGCTTGGTGCAGGTCGGGAACTGATTGGCTGACACCGCCAC-
GTGTCTTATCATGGTTGGCTTGTGAGCAGTGATCGGA--TCCCGCCTTGCATCAACTGAATCGGAGCCCGAGGTGAAAAGAAGAAGAAACCTAAAAAAA-GGATCT-AAC---TTTCAGATC--
TGACTCAATCTTTGTTCCTCTT-------------------AAGGTATAATCTTGAAATCAATCTTG---TTTATGTT------
>Rau-1_*MIR168b*
ATTAAATACCAAGCA-AAGAAGGC------------------TA----TTTGATATAAAGTCT-AAATCAGAGAAAA-TA-TCTTTACCAGATAT--------------GGTAACTGTTTTCTCATTCTTC---------TCTTACATCTT--
TTTCAATAAGAGAGGAAAAAAAAAAAG----------------------TACGATGAT---GATA-------AAG-TTACCGGCGGTCTCTGATTCGCTTGGTGCAGGTCGGGAACTGATTGGCTGACGCCGACAC-
ATGTGTTGTTATGGTTGGCTTGTAAGC------------TCCCGCCTTGCATCAACTGAATCGGAGCCCGAGGTGAAAAAAATTT---------------GGATCC-AAC---TTTCAGATC--TGAGTCATTCTTCTTC-ACGCTT-----
-------------AAGGTATAATCTTGAT-------------ATTTTGTTT---AT
>Tsa_*MIR168b*
GTTAAA-ACCCATCA-AAGGAGGC---TAATTAATTCTCGATA----TTTGATAG-------------AGAGATAAAAA-TCTTTGCCAGATAT--------------GGCAGCTGTTT-CTCGTTCTCTG------TTCTCTTTATCTT-
TTTGCAATGAGAGAGAGAGATAGAGGAA---------------------TACGATGATGATGATA-------AAG-TTACCGGCGGTCTCGGATTCGCTTGGTGCAGGTCGGGAACTGATTGGCTGACACCGCCAC-

GTGGCTTGCCATGTTTGGCTTGTGAGCAGGGATCGGA--TCCCGCCTTGCATCAACTGAATCGGAGCTCGAGGTGAAAAGAAAGAAAGGAAAA-------GGATCT-AAC---TTTCAGATC--TGACAC-----------
AATCTT--------------------GTTTTAAGTTTTTT---------------TTTGTTTGAAAA
