# Supplementary material for: Evolution of MIR168 paralogs in Brassicaceae
Source: BMC Evol Biol. 2009 Mar 23;9:62. doi: 10.1186/1471-2148-9-62 (PMC2664809; doi:10.1186/1471-2148-9-62)
Supplement: Additional file 3 — Primers. List of primers used for amplification of MIR168 homologs from Brassicaceae. [file 1471-2148-9-62-S3.pdf]

MICROSYNTENIC PHYLOGENETIC FOOTPRINTING OF *MIR168* LOCI IN BRASSICACEAE

Silvia Gazzani, Mingai Li, Silvia Maistri, Eliana Scarponi, Michele Graziola, Enrico Barbaro, Jörg Wunder, Antonella Furini, Heinz Saedler and Claudio Varotto

Additional File 3

A) Primers used to isolate the *MIR168a* and *MIR168b* intergenic regions from Brassicaceae species. “Upstream” and “downstream” refer to transcription direction.

Gene	Primer	Description	Sequence 5' to 3'
MIR168a/b	K313	from mature miR168 towards upstream sequences	AATTAGTTCCCGACCTGCACCAAGC
	K353	from mature miR168 towards downstream sequences	GCTTGGTGCAGGTCGGGAACTAATT
MIR168a	K315	from upstream gene towards mature miR	CCAAAGACCAACATCACAGTTCCTA
	K355	from downstream gene towards miR	TTGGTTCAGGTTCTCAAGCTATGG
	MaLoopStar_Cons1	from miR168a* towards upstream sequences	CGATTCAGTTGATGGAAGGCGGGA
	g014	from miR168a upstream IR[(a)] towards mature miR	GTACAGATCACGCTCACATGG
MIR168b	K320	from upstream gene towards mature miR	GCTCTCTTCACAGTGGTTGGTATCTC
	5453-6s	from upstream gene towards mature miR	CGAGTCTTTAACACCGCTGGCTC
	K359	miR168b from downstream gene towards miR	CACTTCTCCTTCTTCACATATCGTCA
	MbLoopStar_Cons1	from miR168b* towards upstream sequences	CGATTCAGTTGATACAAGGCGGGA

[(a)] IR= intergenic region

B) Primers used for *MIR168a* and *MIR168b* expression studies.

**Gene**	**Primer**	**Description**	**Sequence 5' to 3'**
MIR168a	Mac-P1_For	Forward primer for *pMIR168a::GFP-GUS* construct	CACCATGCTTGTGATGGTTCGATTC
	Mac-P1_Rev	Reverse primer for *pMIR168a::GFP-GUS* construct	AGCCCGATGGTGAGACTCTACTA
MIR168b	Mbc-P1_For	Forward primer for *pMIR168b::GFP-GUS* construct	CACCTCTCTTCACAGTGGTTGGTATCTC
	Mbc-P1_Rev	Reverse primer for *pMIR168b::GFP-GUS* construct	GGTACCGAGACCGCCGGTAACTTTAACAATC
	Mb_RACE_1Rev	*MIR168b* RACE primer	CGAGACCGCAGGTAACTTTAAC
	Mb_RACE_2Rev	*MIR168b* RACE nested primer	CTTTAACAATCATCGCATCTCTC

C) Primers used for phylogenetic reconstruction.

**Gene**	**Primer**	**Description**	**Sequence 5' to 3'**
ITS	ITS-5_For	Forward primer for *ITS*	GGAAGTAAAAGTCGTAACAAGG
	ITS-2_Rev	Reverse primer for *ITS*	GATATGCTTAAACTCAGCGGG
EIF3E	EIF3E_For	Forward primer for *EIF3E*	TTTGAATGTGGCAACTAYTCTRGTGCTGC
	EIF3E_Rev	Reverse primer for *EIF3E*	ACCTCTTCACACTCYYTCATCTT
