# Supplementary material for: Evolution of MIR168 paralogs in Brassicaceae
Source: BMC Evol Biol. 2009 Mar 23;9:62. doi: 10.1186/1471-2148-9-62 (PMC2664809; doi:10.1186/1471-2148-9-62)
Supplement: Additional file 4 — GenBank accession numbers. List of GenBank accession numbers corresponding to the sequences obtained during this study or downloaded from public databases. [file 1471-2148-9-62-S4.pdf]

MICROSYNTENIC PHYLOGENETIC FOOTPRINTING OF *MIR168* LOCI IN BRASSICACEAE

Silvia Gazzani, Mingai Li, Silvia Maistri, Eliana Scarponi, Michele Graziola, Enrico Barbaro, Jörg Wunder, Antonella Furini, Heinz Saedler and Claudio Varotto

Additional File 4

GenBank accession numbers corresponding to *miR168a*, *miR168b* and *AGO1* sequences.

Ath *MIR168a*
AL022580.1 (AT4G19395)

***MIR168a* from Brassicaceae species**
AM900024-AM900039

Ath *MIR168b*
AB020744.1 (AT5G45307)

***MIR168b* from Brassicaceae species**
AM900001-AM900016

Ath *ITS*
X52320

***ITS* from Brassicaceae species**
AM905711-AM905726

Ath *EIF3E*
AL137080.2 (AT3G57290)

***EIF3E* from Brassicaceae species**
AM889288-AM889304

Aly *MIR168a* assembly

gnl|ti|1511654458 name:FAFG16900.b1 mate:1511651866
gnl|ti|1511795285 name:FAFG544.x4 mate:1511795667
gnl|ti|1547512654 name:FAFG370062.x1 mate:1547512271
gnl|ti|1511894322 name:FAFG89347.b1 mate:1511895474
gnl|ti|1512556869 name:FAFH40529.g1 mate:1512561093
gnl|ti|1543071509 name:FAFH247325.g1 mate:1543073141
gnl|ti|1511779788 name:FAFG7608.y2 mate:1511783613
gnl|ti|1511986023 name:FAFG191933.x9 mate:1511982215
gnl|ti|1512339410 name:FAFG268605.b1 mate:1512338738
gnl|ti|1536807956 name:FAFH169683.b1 mate:1536810452

Aly *MIR168b* assembly

gnl|ti|1512490040 name:FAFG319560.y9 mate:1512489664
gnl|ti|1512576453 name:FAFG309013.x20 mate:1512580003
gnl|ti|1512511218 name:FAFH20867.b1 mate:1512511698
gnl|ti|1536807093 name:FAFH167860.b1 mate:1536806805
gnl|ti|1541919515 name:FAFH204722.b1 mate:1541922299
gnl|ti|1541952329 name:FAFH265856.b1 mate:1543086584
gnl|ti|1541967113 name:FAFI114464.g1 mate:1541965097
gnl|ti|1543126764 name:FAFH289236.b1 mate:1543128684
gnl|ti|1549999203 name:FAFI75614.g1 mate:1549999779
gnl|ti|1510500343 name:FAFI25282.b1 mate:1510499671
gnl|ti|1511856949 name:FAFG80385.x9 mate:1511859998
gnl|ti|1510538850 name:FAFI33916.g1 mate:1510540194
gnl|ti|1512140542 name:FAFG206076.y9 mate:1512137111
